# The family of DOF transcription factors in *Brachypodium distachyon*: phylogenetic comparison with rice and barley DOFs and expression profiling

**DOI:** 10.1186/1471-2229-12-202

**Published:** 2012-11-05

**Authors:** Sara Hernando-Amado, Virginia González-Calle, Pilar Carbonero, Cristina Barrero-Sicilia

**Affiliations:** 1 Centro de Biotecnología y Genómica de Plantas (UPM-INIA). Escuela Técnica Superior de Ingenieros Agrónomos, Universidad Politécnica de Madrid. Campus de Montegancedo, Pozuelo de Alarcón, Madrid, 28223, Spain

## Abstract

**Background:**

Transcription factors (TFs) are proteins that have played a central role both in evolution and in domestication, and are major regulators of development in living organisms. Plant genome sequences reveal that approximately 7% of all genes encode putative TFs. The DOF (DNA binding with One Finger) TF family has been associated with vital processes exclusive to higher plants and to their close ancestors (algae, mosses and ferns). These are seed maturation and germination, light-mediated regulation, phytohormone and plant responses to biotic and abiotic stresses, etc. In *Hordeum vulgare* and *Oryza sativa*, 26 and 30 different *Dof* genes, respectively, have been annotated. *Brachypodium distachyon* has been the first Pooideae grass to be sequenced and, due to its genomic, morphological and physiological characteristics, has emerged as the model system for temperate cereals, such as wheat and barley.

**Results:**

Through searches in the *B. distachyon* genome, 27 *Dof* genes have been identified and a phylogenetic comparison with the *Oryza sativa* and the *Hordeum vulgare* DOFs has been performed. To explore the evolutionary relationship among these DOF proteins, a combined phylogenetic tree has been constructed with the Brachypodium DOFs and those from rice and barley. This phylogenetic analysis has classified the DOF proteins into four Major Cluster of Orthologous Groups (MCOGs). Using RT-qPCR analysis the expression profiles of the annotated *BdDof* genes across four organs (leaves, roots, spikes and seeds) has been investigated. These results have led to a classification of the *BdDof* genes into two groups, according to their expression levels. The genes highly or preferentially expressed in seeds have been subjected to a more detailed expression analysis (maturation, dry stage and germination).

**Conclusions:**

Comparison of the expression profiles of the Brachypodium *Dof* genes with the published functions of closely related DOF sequences from the cereal species considered here, deduced from the phylogenetic analysis, indicates that although the expression profile has been conserved in many of the putative orthologs, in some cases duplication followed by subsequent divergence may have occurred (neo-functionalization).

## Background

Transcriptional control is the single most important regulatory mechanism in all organisms. It ultimately depends on transcription factors (TFs) that recognise *cis*-regulatory elements in the promoters of their target genes. It has become evident that TFs are essential components in the regulation of many traits in plants, including some of agronomic importance such as yield or response to biotic or abiotic stresses and to hormones. TFs have played also a central role in crop domestication and in the evolution of plants [1,2]. Molecular genetic studies have so far identified major domestication genes in plants, and many of them encode TFs [3], including the rice *shattering* genes *sh4* and *qSH*[4,5], the *Teosinte Branched1* (*Tbr1)* gene, first described in maize, which affects plant architecture [6], and the *AP2-like* wheat gene *Q*, which is involved in the inflorescence structure [7].

The availability of plant genome sequences has made a great impact on plant biology, defining the protein-coding content of several species and illustrating how these have increased in complexity during the evolution of higher plants [8-10]. Complex organisms contain a large number of TFs. In plants, ≈ 7% of all genes encode putative TFs [11]; by genome-wide identification, the Arabidopsis and the rice genomes have been predicted to contain at least 1,600 TF genes [12,13] and over 2,000 TF genes in the *Brachypodium distachyon* genome [10].

The TF proteins are composed of at least two discrete domains: a DNA binding domain, which interacts with *cis*-regulatory elements in the promoters of their target genes, and an activation or repression domain. TFs operate in complex networks based on protein-protein interactions to regulate many physiological and biochemical processes by modulating the rate of transcriptional initiation. This combinatorial control, involving both transcriptional activators and repressors, integrates signals and results in diverse outcomes of gene expression. TFs are classified into families, mainly on the basis of their conserved DNA binding domains, and both, the number of families, as well as, the number of members in each family have increased in the course of evolution [14].

The DOF (DNA binding with One Finger) transcription factor family belongs to the class of zinc finger domains and it is characterized by a binding domain of 52 amino acid residues that is structured as a Cys2Cys2 (C2/C2) zinc finger [15] that binds specifically to *cis*-regulatory elements containing the common core 5’-T/AAAAG-3’ [16]. The family has evolved from a common ancestor in *Chlamydomonas reinharditii,* where only one *Dof* gene has been found, and expanded into the different taxonomic groups from ferns and mosses to vascular plants [17]. DOF TFs are not found in other eukaryotes such are yeast, Drosophila, Caenorhabditis or humans. The number of *Dof* genes varies depending on the species; bioinformatic analysis of the Arabidopsis and rice genome predicts 36 and 30 *Dof* genes respectively [18], while 26 have been described in barley [17], 31 in wheat [19], and 28 in sorghum [20].

Since the identification of the first DOF protein, ZmDOF1, from maize, that acts as a transcriptional activator of the light regulated *C4 Phospho-Enol-Pyruvate-Carboxylase (PEPC)* gene [21,22], *Dof* genes have been extensively studied from various plant species. Generally, DOF proteins are reported to participate as transcriptional regulators in many plant-specific biological processes, such is the case of CDF1, 2, 3 and 5 DOF proteins from *A. thaliana* that regulate the photoperiodic flowering time by repressing the *CONSTANS* gene [23-25]. HPPBF3, COG1 and OBP3 are three DOF proteins that participate in the signalling pathways mediated by phytochrome A and B and Cryptochrome 4 [26-28]. The OBP binding proteins (OBP 1–4) interact with the *ocs* stress-response element in plant promoters [29,30]. Other DOF transcription factors such as AtDOF5.6/HCA2 regulates inter-fascicular cambium and vascular tissue formation [31], and AtDOF4.7 is expressed in the abscission zone of flowers and participates in the expression of cell wall hydrolytic enzymes [32]. In Arabidospsis, the DOF proteins DAG1 and DAG2 influence, with opposite effects, seed germination [33,34], and DOF6 has been recently characterized as a negative regulator of seed germination that interacts with TCP14 [35].

The *Nicotiana tabacum* NtBBF*,* is induced by auxin and acts as a regulator of the expression of the oncogene *rolB*[36]. In potato StDOF1 regulates guard cells specific gene expression [37] and StSRF1 modulates the carbohydrate metabolism in the storage roots [38]. PsDOF7 from *Pisum sativum* activates the expression of the chloroplast thioredoxins f and m that are linked to short-term changes in the sugar and thiol status in plants [39]. In the higher plant ancestor *Physcomitrella patens* the PpDof1 acts as transcriptional repressor, controlling nutrient-dependent filament growth [40].

In cereal seeds, DOF transcription factors have been shown to regulate gene expression both during seed maturation and upon germination. In maize, PBF (Prolamin-Binding-Factor) and its orthologs from barley and wheat, HvDof24-BPBF and WPBF, are important activators of genes encoding reserve proteins during endosperm development, and its presence is associated with crude protein content and starch content of barley seeds [41-43]. Besides, BPBF is a transcriptional repressor of gibberellin-responsive hydrolase genes induced in the aleurone layers upon seed germination [44]. Another barley DOF, HvDOF23-SAD, has been shown to activate gene expression both during seed maturation and upon germination [45,46]. Other DOF proteins from barley, HvDOF19 and HvDOF17, mediate the ABA-repression of hydrolase genes in germinating aleurone cells [47]. These DOF regulators interact physically with other proteins belonging to different TF families such as bZIP, MYBR2R3, MYBR1 and WRKY [42,45,47-50]. Other seed DOFs, like GmDOF4 and GmDOF11 from soybean, have been implicated in lipid metabolism and their over-expression in *A. thaliana* increase seed lipid content by activating genes associated with fatty acid biosynthesis [51].

*Brachypodium distachyon* has been the first member to be sequenced within the Triticeae tribe [10], that includes important crops like wheat, barley and oats [52]. Due to its small genome size, short life cycle, and easy transformation, Brachypodium has become a model system for functional genomic studies in temperate cereals.

This paper reports genome wide *in silico* identification of the *Dof* gene family of *B. distachyon*. Phylogenetic comparison with closely related DOF proteins from rice and barley has been done, as well as, an expression analysis with special emphasis during seed maturation and germination.

## Results

### *In silico* identification of *Brachypodium distachyon DOF* encoding genes

**Figure 1:**
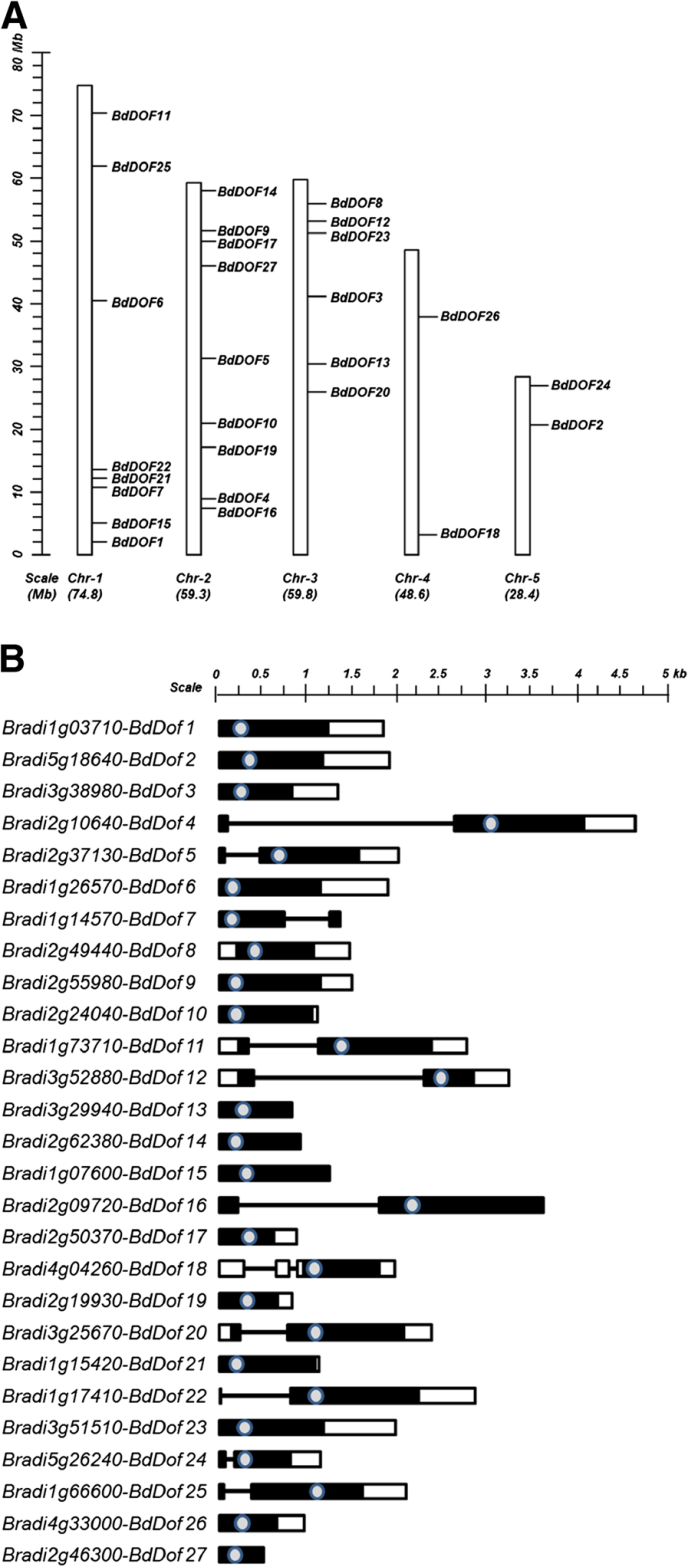
**Chromosomal localization and intron-exon structure of the 27 *BdDof *genes annotated along the five chromosomes of Brachypodium.(A)** The chromosome numbers and their size (Mb) are indicated at the bottom of each bar. **(B)** The intron-exon structure is that predicted by the Brachypodium Genome Annotation Project. Exons are represented by bars: black bars indicate the open reading frames and white bars the non-translated regions. Introns are represented by connecting lines. The Dof domain is represented by a grey circle. The size of exons and introns can be estimated using the horizontal scales.

**Figure 2:**
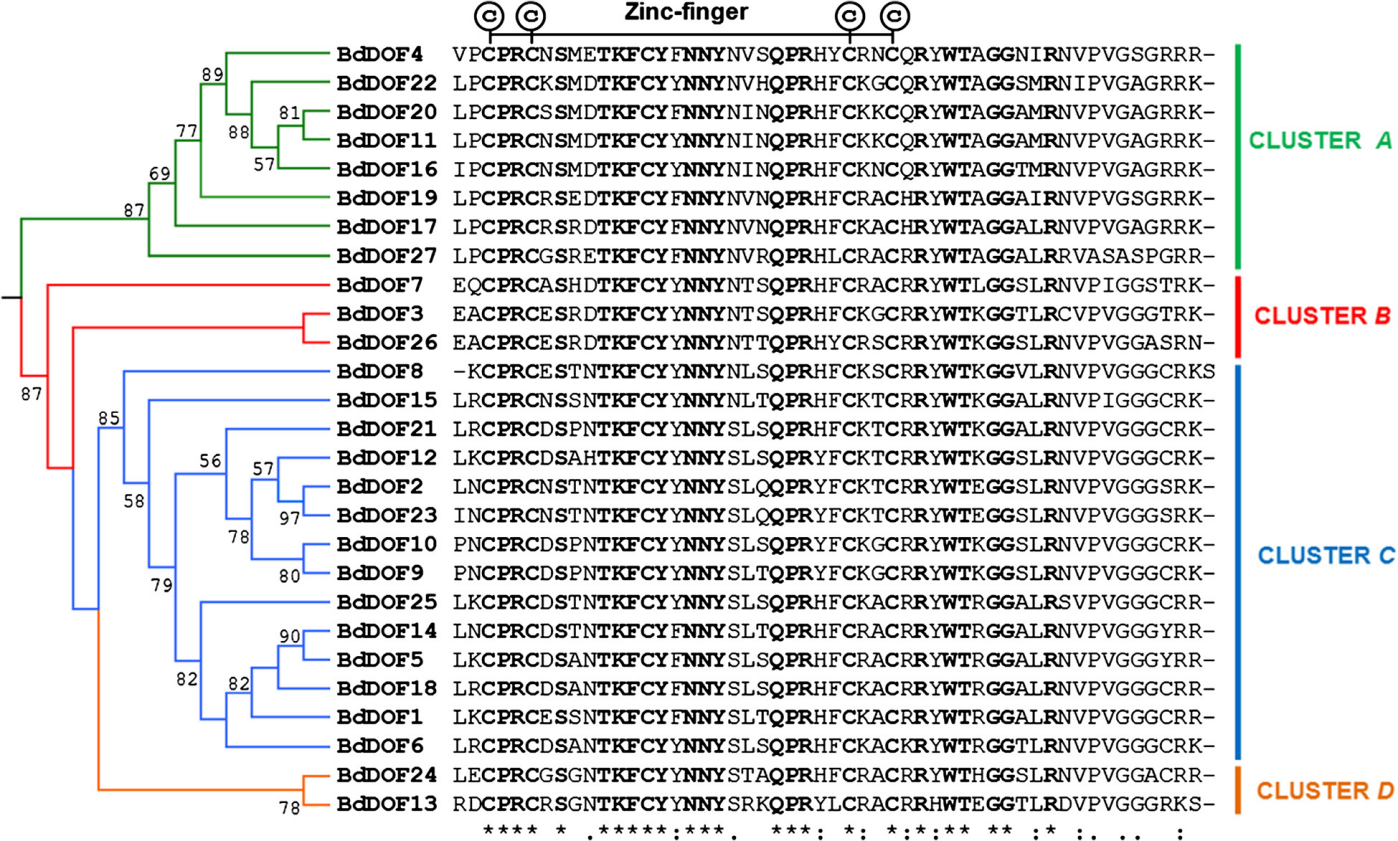
**Deduced amino-acids sequences of the *BdDof *DNA-binding domains and phylogenetic analysis.** The phylogenetic tree has been constructed from a complete alignment of the 52 amino acid residues spanning the classical DOF-binding domain of the predicted 27 DOF proteins in *B. distachyon*. Bootstraping values are indicated as percentages (when > 50%) along the branches. The resulting four gene clusters are indicated in different colours: *A* = green, *B* = red, *C* = blue, *D* = orange. Strongly conserved residues are indicated below the alignment: *asterisks* indicate a perfectly conserved residue; *dots* indicate similar residues and *double dots* indicate that residue variation occurs within weaker conserved residue groups. The four cysteine residues (C) putatively responsible of the zinc-finger structure are indicated above.

To identify the DOF proteins encoded by the *B. distachyon* genome, the consensus amino acid sequence of the DNA binding domain of DOF proteins, previously annotated from barley [17], has been used to perform a BLAST against the whole Brachypodium genome database (http://blast.brachypodium.org/) [10]. Twenty seven non-redundant *Dof* transcription factor genes have been identified (Additional file 1) numbered from *BdDof1* to *BdDof27*, according to their homology to the corresponding barley DOFs. All of them have a typical binding domain of 52 residues spanning a single C2/C2 zinc finger structure (DOF domain). Their schematic distribution along the five Brachypodium chromosomes appears in Figure 1A and their exon-intron structure is shown in Figure 1B. According to their predicted structures, sixteen of the *BdDof* genes have no introns whereas ten of them contain one intron, generally placed up-stream of the DNA binding domain, with the exception of *BdDof7* with an intron down-stream of it, and of *BdDof18*, which has two introns. The highly conserved 52 residues of the binding domain of *B. distachyon* DOFs, has been used to produce a phylogenetic tree (Figure 2) where these proteins are grouped into four Major Clusters *A*; *B; C* and *D*. Clusters *A* and *C* comprise the majority of BdDOF family members (22 in total), whereas the Clusters *B* and *D* contain only 3 and 2 members, respectively. A high bootstrap value, as well as, the comparative analyses of the deduced amino acid sequences of the BdDOF proteins by the MEME software supports this phylogenetic tree [53,54]. The schematic representation of the different conserved motifs can be found in Additional file 2, and the amino acid consensus sequence of each motif in Additional file 3.

### Phylogenetic comparison of DOF proteins from Brachypodium, barley and rice

**Figure 3:**
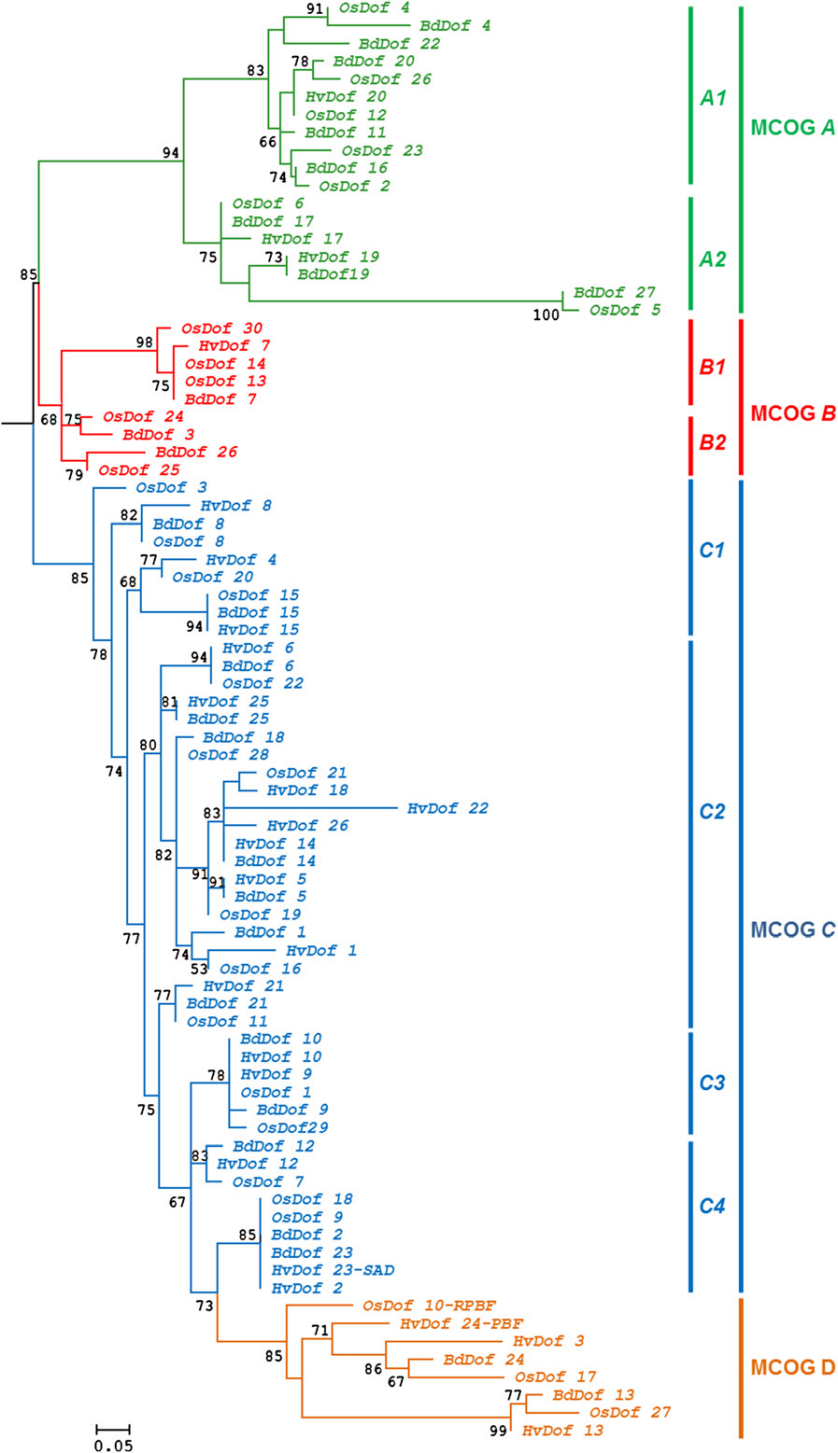
**Joined phylogenetic tree of the Brachypodium, barley and rice DOF transcription factors.** An unrooted tree is shown for a homologous region of 52 amino acids that spans the predicted DOF domain sequences of the 81 Brachypodium, barley and rice *Dof* genes. Bootstraping values are indicated as percentages (when >50%) along the branches. The resulting Major Clusters of Orthologous Genes (MCOG) are shown in different colours: *A* = green, *B* = red, *C* = blue, *D* = orange. The scale bar corresponds to 0.05 estimated amino acids substitution per site.

**Figure 4:**
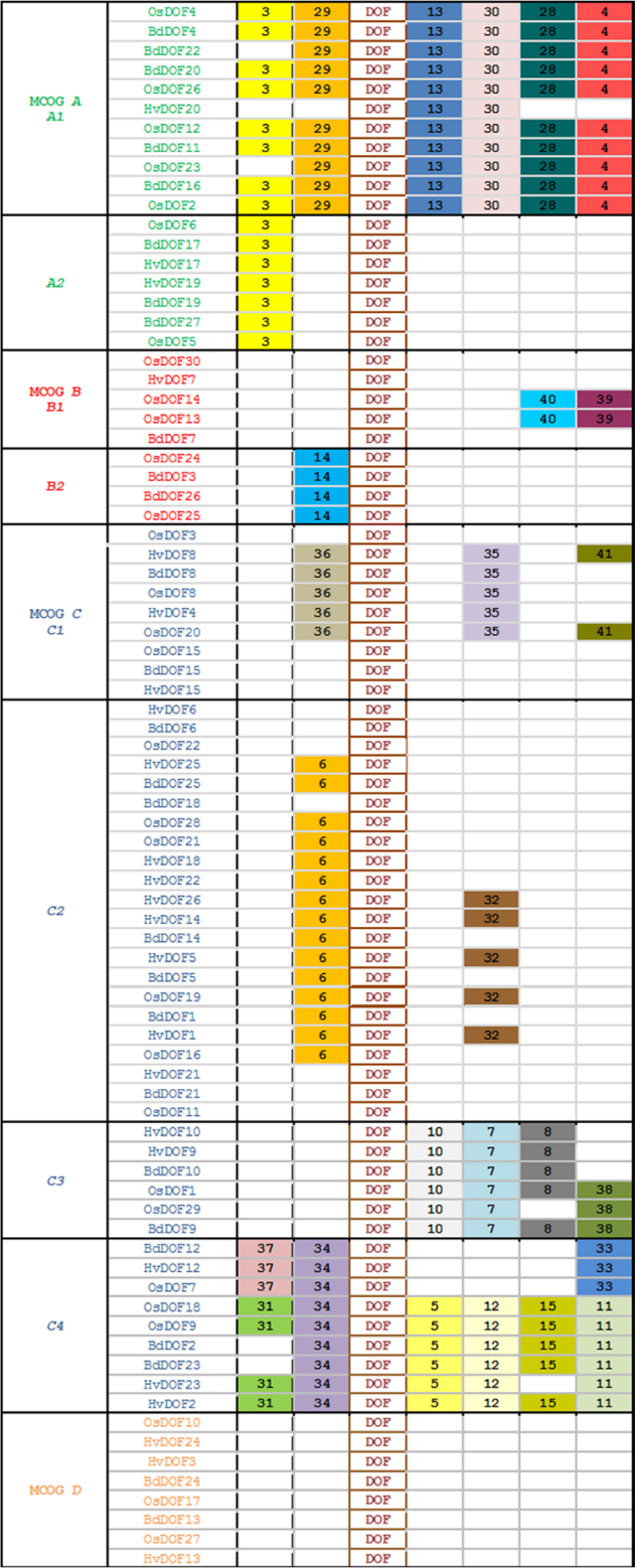
**Distribution of the conserved motifs along the DOF transcription factors clustered in the MCOGs.** Motifs have been identified by means of the MEME software using the deduced amino acid sequences of the 81 Brachypodium, rice and barley *Dof* genes listed in Figure 3. Position of the identified motifs is relative to the Dof domain. Multilevel consensus sequences for the MEME defined motifs are listed in Table 1.

In order to gain insight into the evolutionary relationships among the DOF proteins from Brachypodium, rice and barley, a combined phylogenetic tree has been constructed (Figure 3) where these proteins are grouped into four Major Clusters of Orthologous Groups (MCOG *A*; *B*; *C* and *D*) subdivided into subfamilies. Several putative orthologs (i.e. BdDOF12/HvDOF12/OsDOF7 or BdDOF15/HvDOF15/OsDOF15) and paralogs (BdDOF2/BdDOF23 or BdDOF11/BdDOF20/ BdDOF16) have been identified. This phylogenetic tree is further supported by the comparative analyses of the deduced amino acid sequences of the whole DOF proteins by the MEME software (Figure 4 and Table 1). The majority of the conserved motifs are found in a given subfamily, with the exception of motif 3 that is found both in subfamilies *A1* and *A2* of MCOG *A*. The *D* family members have only in common the DOF binding domain.

**Table 1:** Group and sub-group of consensus specific conserved amino acids motifs

**Motif**^**a**^	**E-value**	**Multilevel consensous sequence**^**b**^
**1**	3.3e-3898	CPRCDST[ND]TKFCY[YF]NNY[SN]LSQPR[HY]FC[KR][AT]CRRYWT[KRA]GG[AS]LRNVPVGGG[CR]R[KR]
**3**	1.0e-081	[GAD][DA][GAS][GLA][IF]KLFG[KR][VT]I[PT][LVP][PQ]
**4**	1.9e-054	[ALP][PRL][VAFL][LM][QHK][AG]NP[AV]A[LF][ST]RS[QV][ST]FQE
**5**	2.0e-040	GA[FL]SAMELLRSTGCY[MV]PL[PQ]Q
**6**	7.7e-148	[PS][MG]SM[ST]ERAR[LM]A[RK][VI]P[QL]PEPGL[KN]
**7**	2.8e-043	[DK][PV][AP][AT][AADGSTIDLA[ML]LY[SA]KFL[SN][HN]Q
**8**	5.4e-034	LGEL[NA]F[AGSV][MV]DQSC[FY]D[SA]LGLP[TAM][DP]
**10**	6.5e-027	[HP]GP[VL]RPDM[VL]LEGMVGN
**11**	7.1e-027	R[LM]LFPFEDLKP
**12**	9.0e-026	[HG][EG][GA]A[HQ]DLNLAFPHH[HG]
**13**	3.3e-024	[WM][PV][NPA][GT]AW[NS][ALS]PW[PI]
**14**	2.7e-011	[PQ][QP]FA[GT]VDLRRPKGY
**15**	3.1e-012	[EDQ][FY][MAPT]AFPSLESSS[VIM][CG][NG]
**28**	7.2e-209	[GEKR][DE][DE][KN][GER][EN][GKN][KS][LV]W[VI]PKT[LI]RID[DN][PA][DA]E[AV]A[KR]SSI[WR][SAT][TL][LFI]GI[KE][PG][DG][DK][RKPV]G[IM] [FD][KR][SPG][FR][QG][SC][KG]
**29**	2.9e-074	[KQ]T[EAQ][DGNS][DE][EGT][SA][SDN][QE][DEK][KE][VK]LKKPDKILP
**30**	1.1e-030	S[PSI][VST][CS][ST]M[SN][SGN][SC][FP][TV]LGKH[PS]R[DE][GS]D[]
**31**	6.2e-036	MDAA[HQ]W[HP]QGLGLVKPMEEM[LI]M
**32**	2.8e-027	[NM][G][LM]EQWR[AL][AQ]QMQSFPFFHAMDHQ
**33**	7.3e-026	[AG]HHHHGSSA
**34**	2.1e-030	R[RK][AL]RPQ[KH][ED][KQ][AP][LI]
**35**	6.2e-027	[STI][TFQ][AFNPT][NSTY][PAS][FDV][ADP][AGPT][DLP][VLS][PQ]PPAP[IM]FADQA[AT][AT][LF]ASLF[AG][PT]P[PR]P[PA][PF][LST][PFQ][VAS] [FAL][SNPQ][FAR]
**36**	2.6e-025	MQ[ED]F[QH][SP][IV]PGL[AT]GRLFGGAA[AD][AR][ADP][AID][RGIV][RAL]
**37**	1.2e-016	PMH[FI]CMDSDWLKG[IM]V
**38**	1.7e-036	LSSWCSIVPSLSTWEEPKYDSLDSFPDD[AT][ML]SLH[DE][CGH][GIM][IL]
**39**	8.9e-015	RVADHQHDDGRRAVRRGDVRALRRRRLPAHGPFVGTVVAAVTVVVVRCNL
**40**	2.8e-014	EEWMQEQDGLLCMRGRRCGRRGGCLPRPRDWFAALLAADPAAAAVTRDQ
**41**	2.3e-011	TVADM[AT]PF[MT]SLDAGIFELGD[AV][PS]PA[AD]YWN[AG]GSCWTDV[PQ]DP[NS]VYL

^a^ Numbers correspond to the motifs described in Figure 4. ^b^ Consensus amino acid sequences obtained from the analysis of the 81 Brachypodium, rice and barley Dof genes with the MEME software. Motifs from 1 to 15 are equivalent to the motifs 1 to 15 described in Additional files 2 and 3. Motif 1 corresponds to the DOF DNA-binding domain.

### Expression of the *BdDof* genes in the major organs of *B. distachyon*

**Figure 5:**
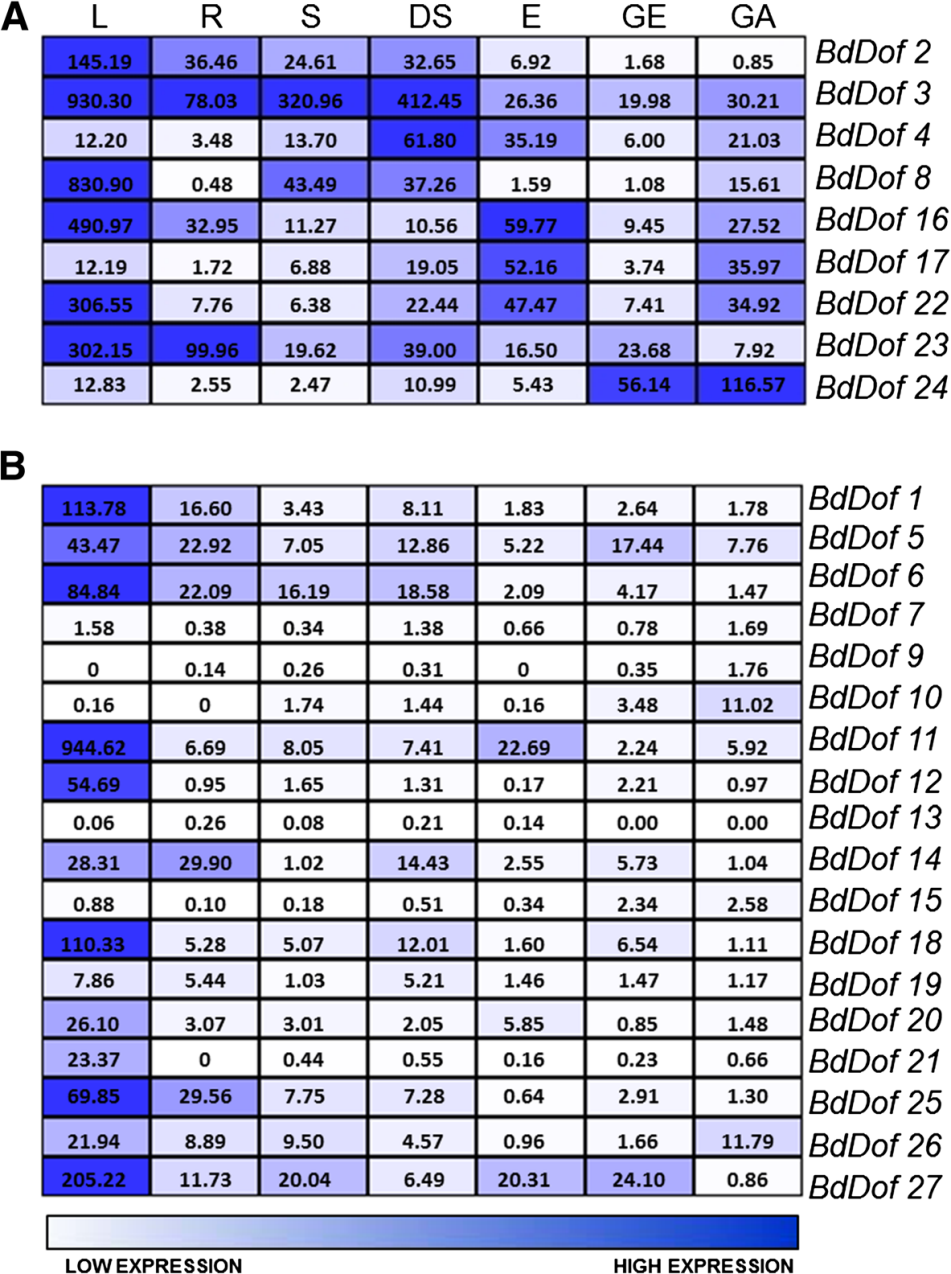
**Expression levels of the *BdDof *genes in various organs of the *Brachypodium distachyon *plants.** Expression levels are measured by RT-qPCR in leaves (L), roots (R), spikes (S), developing seeds (DS), dry embryos (E), germinating embryos (GE) and de-embryonated germinating seeds containing the aleurones (GA). **(A)** Expression pattern of the 9 most abundant *Dof* genes in seeds; **(B)** expression pattern of the 17 less abundant *Dof* genes in seeds. The number inside the square indicates the relative expression levels standardized to the constitutive *BdGAPDH* gene expression (%)*.* Values are the mean of at least three independent experiments.

To study the expression of the annotated *BdDof* genes, RT-qPCR analyses have been done in several organs: leaves (L: a mixture of young and mature leaves, 12 and 40-day-old), roots (R: 12-day-old), pre-anthesis spikes (S), developing seeds (DS: a mixture from 2 to 12 days after pollination, dap), dry embryos (E), and germinating seeds (a mixture from 8 to 96 hours after imbibition, hai). In this latter case, germinating embryo (GE) and the de-embryonated seed containing the germinating aleurone (GA) have been processed separately. For this purpose, a set of gene-specific primers from the 3’-non coding regions of the *BdDof* genes have been designed; to ensure reliability of the data, primer pairs that displayed PCRs efficiencies between 2 ± 0.1 have been selected (Additional file 4) and the expression values compared to that of the constitutive *BdGAPDH* gene (Glyceraldehyde 3-Phosphate Dehydrogenase) [55]. A colored schematic representation has been done (Figure 5) using the TM4 software [56]; in Additional file 5 the expression values of the *BdDof* genes (mean ± standard error) can be found. According to their expression profiles, the genes have been divided into two groups: the 9 genes with the highest seed expression values are grouped in Figure 5A; in Figure 5B, the global expression of the 18 remaining *BdDof* genes are included.

Among the genes preferentially expressed in seeds (*BdDof4*, *BdDof17* and *BdDof24*), *BdDof4* presents their maximum transcript levels in the developing seeds, but it is also abundantly expressed in dry embryos and can be detected, although moderately, in the de-embryonated germinating seeds (GA). Transcripts of *BdDof17* are especially abundant in dry embryos and faintly detected during seed maturation. The most abundant transcript in germinating seeds is that of *BdDof24,* both in the embryos and in the rest of the seeds*. BdDof3* is ubiquitously expressed and its transcripts are abundant in leaves, pre-anthesis spikes and in the maturation phase of seeds. Genes *BdDof2*, *BdDof8*, *BdDof16*, *BdDof22* and *BdDof23* are expressed preferentially in leaves, and *BdDof2*, *BdDof16* and *BdDof23* are also abundant in roots.

The *BdDof* genes appearing in Figure 5B are poorly expressed in seeds. However twelve of them are present in leaves, and four of these: *BdDof5*, *BdDof6*, *BdDof14* and *BdDof25* are also found in roots (>20% of the *BdGAPDH* expression). The transcripts of the *BdDof7*, *BdDof9*, *BdDof10*, *BdDof13*, *BdDof15* and *BdDof19* are faintly detectable in all the organs analyzed.

### Expression profiles of the *BdDof* genes during seed development

**Figure 6:**
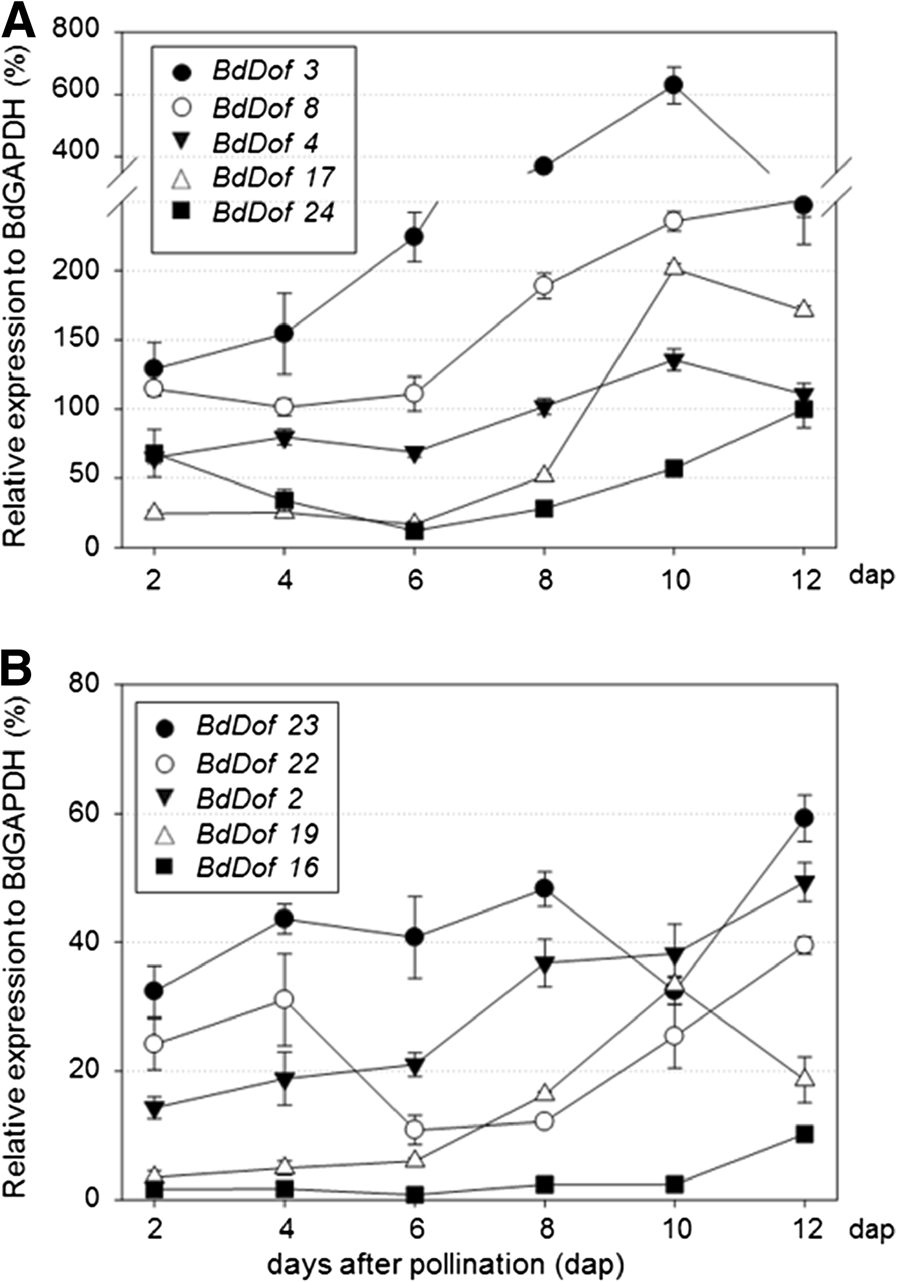
**Expression kinetic profiles of the most abundant *BdDof *genes during seed maturation.** Seeds at different days after pollination (2, 4, 6, 8, 10 and 12 dap). Relative transcript levels are standardized to the constitutive *BdGAPDH* gene expression (%). Values are the means ± SE of at least three independent experiments. The scale on the Y axis in **(A)** or **(B)** varies depending of the expression level of the genes analysed.

**Figure 7:**
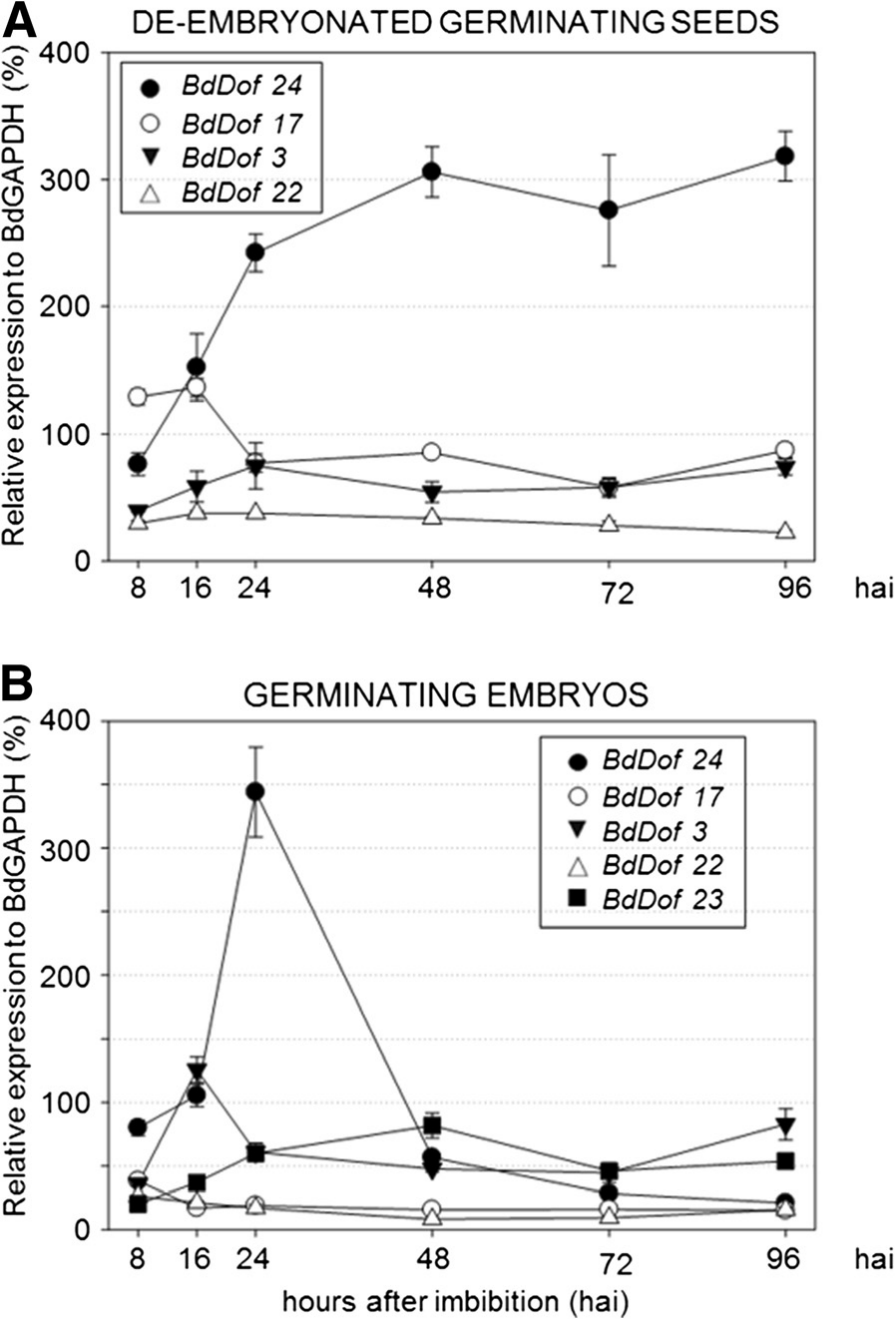
**Expression kinetic profiles of the most abundant *BdDof *genes expressed in germinating seeds.(A)** De-embryonated seeds containing the aleurone and **(B)** embryos from germinating seeds at different hours after imbibition (8, 16, 24, 48, 72 and 96 hai). Relative transcript levels are standardized to the constitutive *BdGAPDH* gene expression (%). Values are the means ± SE of at least three independent experiments. The scale on the Y axis in **(A)** or **(B)** varies depending of the expression level of the genes analysed.

A more detailed expression profile for the genes included in Figure 5A has been further investigated in the two phases of seed development: maturation and germination. Seeds have been collected at different maturation stages (2, 4, 6, 8, 10 and 12 dap), and in germinating seeds at different hours after imbibition (8, 16, 24, 48, 72 and 96 hai); in this case the embryos have been processed separately from the rest of the seeds. The expression profile for the *BdDof* genes analysed is shown in Figures 6 and 7.

The most abundant transcripts in seeds during maturation are those of *BdDof3,* with a sharp peak of expression at 10 dap (600% that of *GAPDH)*. Similarly, the expression of the genes *BdDof8*, *BdDof4* and *BdDof17* increases during development, whereas *BdDof24* presents a high expression level at the beginning and at the end of the maturation period considered (2 dap and 12 dap), decreasing to low levels at the middle stage (Figure 6A). The less abundant transcripts of this group are those of genes *BdDof23*, *BdDof22, BdDof2, BdDof19* and *BdDof16*, their expression moderately increasing with seed maturation with the exception of *BdDof19* that has a peak at 10 dap and decreases thereafter (Figure 6B).

In Figure 7, the expression kinetics in the embryos and in the de-embrionated (containing the aleurones) germinating seeds of *BdDof24*, *BdDof17*, *BdDof3, BdDof22*, and *BdDof23* are presented. The most abundant transcripts in both cases are those of *BdDof24*, but whereas in de-embrionated germinating seeds (GA), the transcript levels increase progressively with time of imbibition reaching a plateau value ( ~300% that of *GAPDH*) from 48 hai to 96 hai, in germinating embryos an expression peak is reached at 24 hai that sharply decreases after 48 hai. In germinating seeds, the transcripts of *BdDof2*, *BdDof4*, *BdDof8*, *BdDof16* and *BdDof19* are barely detected (data not shown).

## Discussion

Transcriptional regulation is an important mechanism underlying gene expression; the number, position and interaction between different *cis*-elements and the TFs at a given gene promoter determine the gene expression pattern. These TFs can be classified into gene families according to the presence of a particular DNA-binding domain. In this study, we have conducted a comprehensive search to identify the family of *BdDof* genes in the *Brachypodium distachyon* genome database. A complete overview of this gene family in Brachypodium is presented, including a multiple sequence alignment, the intron-exon structures, a phylogenetic comparison with closely related cereal species and its expression in different organs.

The overall intron-exon structure indicates that, the majority of *BdDof* genes are intronless, as observed in other species, or have one intron, as occurs with five members of Cluster *A*; exceptionally *BdDof18* in Cluster *C* have two introns [17,18]. Multiple sequence alignment of these BdDOF proteins with the 52 amino acid residues spanning the DOF binding domain have defined four groups of paralogous genes in Brachypodium, that share common proteins motifs outside the DOF domain, detected in the MEME analysis. The comparative phylogenetic analysis of these BdDOF proteins with those from rice and barley, have defined four MCOGs.

RT-qPCR analysis has shown that the majority of *BdDof* genes are expressed in leaves, although nine of them are highly (or exclusively) expressed in seeds. No similarities in expression patterns are generally observed within members of the same Cluster of paralogous genes, although *BdDof2* and *BdDof23* in Cluster *C*, that share at least five motifs outside of the binding domain, are also quite similar in their expression patterns, indicating that probably they arose by a recent gene duplication, followed by a translocation event since they are located in Chromosomes 5 and 3 respectively. Genes preferentially expressed in leaves (approximately ten times more than in the rest of the organs analysed) are those of *BdDof11*, *BdDof27*, *BdDof1*, *BdDof18*, *BdDof12* and *BdDof20*. A similar expression pattern has been found in some of their orthologous partners in rice, using the tool Rice Gene Expression Anatomy Viewer from the Rice Genome Annotation Project [57]. This is the case of *BdDof12* and its orthologous *OsDof7*, and *BdDof1* and *OsDof21*. Preferentially expressed in leaves are genes *BdDof5*, *BdDof6*, *BdDof14*, *BdDof25* and *BdDof26*, although their transcripts are ubiquitous. Genes *BdDof7*, *BdDof9*, *BdDof10*, *BdDof13*, *BdDof15* and *BdDof19* are barely detected in the organs assayed.

Since several studies support a role for DOF TFs in the regulation of genes encoding seed storage proteins during seed maturation as well as of genes encoding hydrolases involved in the mobilization of reserves upon seed germination, we have selected for a more thorough analysis the genes highly expressed in seeds. These are *BdDof2*, *BdDof3*, *BdDof4*, *BdDof8*, *BdDof16*, *BdDof17*, *BdDof22*, *BdDof23* and *BdDof24*. *BdDof19* is also included in the seed time-course study, because it is closely related in sequence to *HvDof19*, an important regulator of hydrolase gene expression upon germination in barley seeds [47]. *BdDof4*, *BdDof17* and *BdDof24* are preferentially expressed in seeds, with very low levels of expression in the vegetative organs analysed. The rice ortholog of *BdDof4* (*OsDof4*) that is expressed in developing seeds, has been described as having a role during the grain filling process [58]. *BdDof24* is highly expressed in seeds, both in the maturation phase and upon germination. Its putative orthologs from barley (*HvDof24BPBF*) and rice (*OsDof10RPBF*), have similar expression patterns and they are transcriptional activators of genes encoding endosperm-specific storage proteins [59,60] and transcriptional repressors of hydrolase genes in the aleurone upon seed germination [44]. Other DOF TFs reported to be important in the regulation of barley hydrolase genes upon germination are those encoding transcriptional repressors *HvDof17* and *HvDof19*[47]. The expression of their putative Brachypodium orthologs shows that *BdDof17* has a predominant expression in dry embryos, as well as in de-embryonated germinating seeds, peaking at the first two stages analysed (8 and 16 hai) and decreasing thereafter, and it is also expressed during maturation. The same analyses for *BdDof19* indicate that although its expression is ten times lower than that of *BdDof17* during seed maturation, its pattern is compatible with a possible role in the regulation of storage protein genes; however, no transcripts are detected in the samples of germinating seeds.

*BdDof8*, *BdDof2* and *BdDof23*, have been included in the group of genes expressed abundantly in seeds. Transcript levels of *BdDof8* increase as maturation progresses and an expression peak is detected in germinating seeds (data not shown). *BdDof2* and *BdDof23* are expressed in the maturation phase of seed development, and only the transcripts of *BdDof23* are detected in germinating embryos. These data suggest that *BdDof23* may have a similar role during the maturation process as its orthologous in barley *HvDof23SAD*[45], and its absence in de-embryonated germinating seeds probably indicates that it does not control the expression of hydrolase genes in the aleurone as opposed to its ortholog in barley [46].

Although not preferentially expressed in seeds, the time course during seed maturation and germination has been studied for the three remaining genes that presented high levels in seeds: *BdDof3*, *BdDof16* and *BdDof22*. The *BdDof3* transcripts are the most abundant in the maturation phase of seed development, peaking at 10 dap and decreasing drastically thereafter, and can also be detected in germinating seeds; however its orthologous gene in barley has not been found among the ESTs and other sequences presently available.

## Conclusions

The sequences of the 27 members belonging to the DOF gene family have been compiled from the *Brachypodium distachyon* genome database, and its inclusion into four clusters is supported by the sequence of the DNA-binding domain, the conservation of different domains outside the DOF domain and their intron-exon structure. A phylogenetic comparison with the barley and rice DOF proteins and a detailed expression profile study in different organs together with comparison with published functions of the barley and rice DOFs proteins, suggest that there is a strong sequence conservation between the DOFs of these monocot species, although some of their functions may have diverged in the course of evolution (see Additional file 6). This works opens the possibility of a further more focused investigation of the functional role of these DOF regulatory proteins.

## Methods

### Database searches for the identification of Dof family members in *Brachypodium distachyon*

A non-redundant compilation of the *Hordeum vulgare* and *Oryza sativa Dof* genes have been collected from two different databases: NCBI (http://www.ncbi.nlm.nih.gov) and TIGR (http:/blast.jcvi.org/euk-blast/), respectively. The amino acid consensus sequence of the DOF domain from barley family members has been used to search for potential *Dof* genes in the genome of *Brachypodium distachyon* through BLASTP at the “Brachypodium Database” (http://www.brachypodium.org). The putative DOF protein sequences of *B. distachyon* are analysed with the Interpro program using the PFAM database (http://pfam.sanger.ac.uk[61]) and their DOF domains deduced.

### Dof protein alignment and phylogenetic analysis

The identification of a homologous region in all the DOF proteins sequences (from barley, rice and Brachypodium) that spans the classical DOF-binding domain has been done through a multiple alignment using CLUSTALW [62]. Since the DOF domains of *HvDof11* and *HvDof26* genes found in the databases are truncated, these genes have not been included in the subsequent analysis. From the deduced amino acid sequences identified, phylogenies have been computed using the Phylogeny.fr platform [63], which uses MUSCLE for multiple alignment with Gblocks for alignment curation and the maximum likelihood PhyML method for tree building using the MEGA software 4.0 [64].

### Identification of conserved motifs

The deduced protein sequences of the 81 DOF genes from *O.sativa, H.vulgare* and *B.distachyon* have been further analyzed by means of the MEME program [65], (http://meme.sdsc.edu/meme4_6_0/intro.html). To identify conserved motifs in these sequences, the selection of maximum number of motifs was set to 50 with minimum width of 8 amino acid residues. Conserved motifs identified by MEME have been scanned using PSORT server (http://www.psort.org/) to find subcellular localization signals.

### Plant material and RNA extraction for expression analysis of the *BdDOF* genes

*Brachypodium distachyon* strain Bd21, a community standard diploid inbred line [66], kindly provided by Prof. Garvin (University of Minesota), has been used in this study. Seed have been germinated in the dark at 22°C for one week and then the seedlings transferred to pots in a controlled-environment growth chamber at 22°C and under a 16h day/8h night photoperiod. Samples from young leaves (12-day-old), old leaves (40-day-old), roots (12-day-old), spikes and seeds, at 2, 4, 6, 8, 10 and 12 days after pollination (dap), and dry embryos have been harvested and used for RNA extraction. After-ripened seeds (storage at 22°C and 30% relative humidity in the dark for 3 months) have been surface sterilized, washed and germinated in water imbibed filter paper at 22°C in the dark and samples collected at different times (8, 16, 24, 48, 72 and 96 hours after imbibition, hai) and used for RNA extraction as described above.

Total RNA from vegetative tissues (leaves and roots), spikes and dry embryos has been isolated using the phenol/chloroform method and precipitation with 2M LiCl [67]. For the isolation of RNA from seeds the standard protocol for Arabidopsis seed RNA isolation has been followed [68]. Genomic DNA in the RNA preparations has been eliminated after a DNAse treatment using the DNAse I, RNAse-free from Roche Diagnostics. First strand cDNA has been synthesized with random hexameres using the High-Capacity cDNA Reverse Transcription Kit according to the manufacture’s recommendations (Applied Biosystems).

### RT-qPCR analysis

The transcript levels of 27 *BdDof* genes have been quantified by RT-qPCR with a 7300-Real Time PCR System (Applied Biosystems) using SYBR-green as the intercalating dye. The analysis has been done using three different biological replicates. The primer pairs used for the RT-qPCR analysis (Additional file 4) have been designed on the 3’-non coding region according to the parameters established on the Primer3Plus program (http://www.bioinformatics.nl/cgi-bin/primer3plus/primer3plus.cgi); the primer gene specificity of the *BdDof* primer pairs has been checked by blasting primer sequences in the Brachypodium data base (http://www.brachybase.org) and confirmed by a single peak in the melting temperature curve of the RT-qPCR analyses. To ensure reliability of the results, PCR efficiency has been determined [69]. To this end, four serial ten-fold dilutions of pooled cDNAs have been made starting from 10 to 0.01 ng/μl; when the expression level was too low, genomic DNA has been used. The raw C_t_ values have been plotted against log-transformed concentrations of the serial dilutions, the corresponding PCR efficiency (E) of one cycle in the exponential phase calculated according to the equation: E= 10^(−1/slope)^. The investigated transcripts have showed high PCR efficiency rates (Additional file 4). Quantification has been standardized to the expression of the *BdGAPDH* (glyceraldehyde 3-phosphate dehydrogenase) gene, that was validated as a suitable reference gene across all the plant samples examined (Additional file 7), calculated using the 2^-∆CT^ method and referred as percentage [55,69].

The expression data from leaves (a mixture of young and adult leaves, 12 and 40-day-old respectively), roots (12 day-old), spikes, developing seeds (a mixture from 2 to 12 dap), dry embryos and germinating seeds (a mixture from 8 to 96 hai) have been analyzed using the TM4 software [56].

## Authors' contributions

SHA has annotated the *BdDof* genes, done the MEME analysis and performed the phylogenetic analysis; VGC has done the RT-qPCR expression analyses. PC and CBS conceived the study, participated in its design, coordination and interpretation of the data. CBS has written the manuscript and PC has financed the study and edited the final text. All authors have read and approved the final manuscript.

## Supplementary Material

Additional file 1 **Deduced amino acid sequences of all the Brachypodium *Dof *TFs annotated.** The DOF domain sequences used for the alignment are highlighted in bold. Click here for file

Additional file 2 **Distribution of the conserved motifs along the BdDOF transcription factors clustered in Figure**2**.** Motifs identified by means of the MEME software using the deduced amino acid sequences of the 27 Brachypodium *BdDof* genes represented in Figure 2. Position of the identified motifs is relative to the DOF domain. Multilevel consensus sequences for the MEME defined motifs are listed in Additional file 3. Click here for file

Additional file 3 **Group and sub-group of consensus specific conserved amino acid motifs.** Numbers correspond to the motifs described in Additional file 2. Consensus sequences obtained from the analysis of the 27 Brachypodium DOF proteins with the MEME software. Motif 1 corresponds to the DOF DNA-binding domain. Click here for file

Additional file 4 **Primer sequences used for RT-qPCR analyses, amplicon length and PCR efficiency.** The corresponding RT-PCR efficiency (E) of one cycle in the exponential phase has been calculated according to the ecuation E=10^(−1/slope)^. Click here for file

Additional file 5 **Expression patterns of the *BdDof *genes in varios organs of the *Brachypodium distachyon *plants.** The numbers indicate the relative expresion levels standardized to *BdGAPDH* (mean ± standard error). Click here for file

Additional file 6 **Joined phylogenetic tree of the Brachypodium, barley, rice and the most important*Dof*genes functionally characterized.** Click here for file

Additional file 7 ***BdGAPDH*expression in different organs (A) and at developmental stage of maturating (B) and germinating (C) seed.** Click here for file
