# Supplementary material for: The family of DOF transcription factors in Brachypodium distachyon: phylogenetic comparison with rice and barley DOFs and expression profiling
Source: BMC Plant Biol. 2012 Nov 5;12:202. doi: 10.1186/1471-2229-12-202 (PMC3579746; doi:10.1186/1471-2229-12-202)
Supplement: Additional file 1 — Deduced amino acid sequences of all the Brachypodium Dof TFs annotated. The DOF domain sequences used for the alignment are highlighted in bold. [file 1471-2229-12-202-S1.pdf]

Additional file 1. Deduced aminoacids sequences for all the Brachypodium *Dof* TFs annotated.

>BdDOF1 Bradi1g03710.1
MPAALMAPPRAPDGSSGSMGGLGSGGGGGGGGGSAVVKAGSMTDRARMAKIPAPEAGLKCPRCESSNTKFCYFNNYSLTQPRHFCKACR
RYWTRGGALRNVPVGGGCRRNKRSSKSSKSSSSSSAAACGVAAGVNTSSSSSATTSSATTGSGIMPALGQMPFFAASLVSGSGGGEGQ
YGVGGGGGLLAGVSRSLGFPGGLMGPMGSQQLDSAVENYHHQLGGGMGGSMEQWRLPPPQMQMQQFPFFGGRGAGPDAMSGMQQLQQQ
MQAGISNYPFEPDAGDGSGEGFAAGQMMGGGGGGGKQVVPGSAGLITQLASVKMEDNPPTAMAREFLGLPAAGSLQFWAGGSGGNNGV
SGGGAGAPGGGSGGWVDRLAGFNSSSSGNIL
>BdDOF2 Bradi5g18640.1
MEEMLMGTTNQQQVQGSNPNLIPPPPSSSPAPGAGGGAMGLVAGAVVAGVGSSERKARPQKEKALNCPRCNSTNTKFCYYNNYSLQQP
RYFCKTCRRYWTEGGSLRNVPVGGGSRKNKRSSSSSSANAASGVSASTSIMASSTSMASKNPKLAHHHEGGGAQHDLNLSFPHHGLHG
GMQQQAADQYMAFPSLESSSIGGAMASGNNGRHGPGAGPLSAMELLRSTGCYMPSLHVPMQMPPAAPGEYGAAAVQGFSLGEFRAAPG
PAQSQSSQSLLGFSLDAHHGGTVGMQGMPQDRAGRMLFPFEDLKPTDASGAGSGAGVENGGRHQYEQAGKEQGDGGTGSHAGHDTAPG
FWNGMIGGGTSW
>BdDOF3 Bradi3g38980.1
MGEHAAAEPGRRPAQQQFAGVDLRRPKGYAAAAAAPAKTPAAAPVAATAAAAAAEGEACPRCESRDTKFCYYNNYNTSQPRHFCKGCR
RYWTKGGTLRCVPVGGGTRKRASSSNTSAAAATVKRQKPSKKRRVAPPEPPAAASSADDVPAPDADAAAKTTTTPPTEAASEITTELV
VPAAAAAEEDSFTDLLQEQGGGDDAVALDLGFSDYASAVGKAAGLGDPYSFEWPPAFDLGACWSGAGFADPDPTGVFLNLP
>BdDOF4 Bradi2g10640.1
MAAIRDRGDAAIKLFGRTIPLLHAAAAATEVVTKLRIDENKNDAVPCVSDKLLNVKETPFCSKNSEDNDLQALSRHGGIMGTDSKSED
TKTESDELDQDKVLKKPDIIVPCPRCNSMETKFCYFNNYNVSQPRHYCRNCQRYWTAGGNIRNVPVGSGRRRNKHASHFRQAMMRHDN
NIAAAEDVPSVIHHLPLPLVAPVLPGPIKENETAKEFGSEVPVCNSMATVLDNGEQKGIHLVPLVSGDNKEEQSCASSAAVLGCSENM
TLDSIVKKESGNVSGYCNGMTLPQSHVQSYPNGPALVFPWSPGWNSIAVMAASQCSTEPVHGLEIAKHSLLSWAPPSMMTAPGICAPV
VPFPLMPPFWSCLPGWPNGTWSSPWPGSNGSPNKITCSENNSPTLGKHSREVADMQEEEKRENTLWIPKTRRIDGTAEATKSSILDTL
GIKHDENGLFKSFQRKVPKNDKTPDSPLTLQANPAAFSRSQSFQERT
>BdDOF5 Bradi2g37130.1
MIFPPASFLDSSSWNDNNQINNNFQRHPHHQQQQVVVAGSASATDGSCHEQLLQPSIMQMQQQQQQLAEGGGHGGAQQAQAAAMAGKP
MSMSERARLARVPLPEQGLKCPRCDSANTKFCYFNNYSLSQPRHFCRACRRYWTRGGALRNVPVGGGYRRHAKRAKQPKQQPATSSSS
APATAPTSAPPAMLPTGNHQQLPSGLLPPLLRLADFDAMSLGSTFVSGAGGMGKPPPLTSSGSVDVPAGCYSLGGGGGAMEQWRSVQQ
IPAGFPFFHAMAAADNHLAPAAPGMFHYLGLDHGGNGNGEVVGEEDNQFHHASATTMPSSKREDGFTRGGSNIISMYGNGDHHHQLNA
GYTSSYNSNTAARGNHLL
>BdDOF6 Bradi1g26570.1
MAAGAGGAAAAAAAVQHAGPAGRVGGGGSSSAGGGAAAPDPRAEALRCPRCDSANTKFCYYNNYSLSQPRHFCKACKRYWTRGGTLRN
VPVGGGCRKNKRSRSSSGGGGRVVSSSSAAAAAAGGGTASSSLPLPPHGSASSALQGLHHGSSSLASLLLGTGGGGDHLGLFHQAMQS
VVSDNAAAYEMHNQQQQHQAQVDQLLGLGYGSHSQIQMNKPWLGHDGAGGLFDGFYAPLLSGCSIVPGLEELHVKAEATAGENHHQHK
KDGEQQQQQSGGSWEQHPNSSSSNVEACNNNIMASEALMAAMNPAAAVSSNAATAPTTVSSSQLMYWGNGGGAPAAWPDIGANCGSSI
ATFF
>BdDOF7 Bradi1g14570.1
MARFLQQQQQLVQPTNQNPNANTAREQCPRCASHDTKFCYYNNYNTSQPRHFCRACRRYWTLGGSLRNVPIGGSTRKRLRPAPQQPLR
HRPPVHFASPPPIPQQSAQSQQGLLGSLFALGGAPLLLEGRVGFDLGLGLPGLAGQLGGLTGSGGGGAGEVGLHSLGLRGGGQSAGPG
PTSSLSAPLLWPSSLFENNGGNNVETWKVSGGGAAATAMWAPAPEFSSAATVPQFSQFVFAGYFAVGPCLVLFTYNYKRKAGRSSSVL
VPNG
>BdDOF8 Bradi3g56380.1
MQDFQSIPGLAGRLFGGAAAAADIRRAQGAASSRCGGGFSQEVVKCPRCESTNTKFCYYNNYNLSQPRHFCKSCRRYWTKGGVLRNVP
VGGGCRKSKRSSSSATPASPTDANNANSKTQRRASSSSRDSNSNSGSTSPTATPSSNTTPITFANPFAGDVPPPAPIFADQAAALASL
FAPPPPPPLPVFNFSPQPKTEGSAISSVAVLLAAAEPEAPTSVSESAAAAADMAPFMSLDSTGIFELGDDASAASYWNAGSCCWGTDV
QDPSVYLP
>BdDOF9 Bradi2g55980.1
MDMLSSHPHEAMLPYVPRPPPSLLVDRRAYKTGAEVAPNCPRCDSPNTKFCYYNNYSLTQPRYFCKGCRRYWTKGGSLRNVPVGGGCR
KNRRGKSSSSSSMRSSSASDSSFSGAGRDAGPFGSHRFGSATGSLRPDMVLEGMVGNNPADPGHDMRTAADASTIDLAMLYSKFLNNQ
QPGNNNAVTPESAGGHVMDDGFDTFSGSSDVLRPGVLAAPGGHFDLSPQDGFGEWSGPLSSAGAAGNPASATSTTTSAMLCTDASVQA
ALGELNFVMDQSCFDALGLPMPAEDGGASSVGNDLSSWCSIVPSLSTWEEPKYDSLDSFPDDALSLHDHGILAADHDWSADCQGLEAL
YMP
>BdDOF10 Bradi2g24040.1
MLSSHCENMLAYAAAAAGRRASVLVDPRRYRPNVEVAPNCPRCDSPNTKFCYYNNYSLSQPRYFCKGCRRYWTKGGSLRNVPVGGGCR
KNRRGSKSSSSARPMADDTAAAARDHGPAPAAFSHRFHGPVRPDMLLEGMVGNPPAQLGQPAPSSANKPAAAADGSMIDLALLYSKFL
SHQPANDVRESVDTSSSGSSSATSPGVQPGSGPAQAQAQHGFGRLSSPATASTEQSETTTMLQCADVRAQALGELAFSVDQSCYDSLG
LPTDGGDLILRSTWDQGAKYEPFDSLPVPEDAMSLHGGVPAGGDDVWSKVLGSQGLEAALCRP
>BdDOF11 Bradi1g73710.1
MGQCRAAAGGGDCLFKLFGKTIPVPADSSGSVVDKDQQHSGSSTAEPKVQENIPGDSTGSPTLPEVVDTDDSCAVKKSSADQEEEQSD
TANQKEKLKKPDKILPCPRCNSMDTKFCYYNNYNINQPRHFCKKCQRYWTAGGAMRNVPVGAGRRKSKSISAASHFLQRIRATLPGDP
LCTPIKTNATVLSFGSDTSTLDLTEQMKHLKDKLIPVTQIKNTDDPSVGSCAEGWAKGEEQNQMNSREEVTADKSTNVAQHPCMNGGT
MWPFSCAPSPAYFTSSVAIPFYPAAAAAAYWGYMVPGAWNTPWPPQSQSQSSSSPNAASPVSTMSSCFQSRKHPRDGDEERDTNRNGK
VWVPKTIRIDDADEVARSSIWSLIGINGDKVGTDDGRGCKITRVFYPKDEAKTTTHRVNNSLPFLKGNPAALSRSVTFQERS
>BdDOF12 Bradi3g52880.1
MMAGAAHPMHFCMDSDWLKGMVVPEDQGGAMGSSSPSSPSDTMIIACPQPMQQHQAQQQDRRLRPQHDSPLKCPRCDSAHTKFCYYNN
YSLSQPRYFCKTCRRYWTKGGSLRNVPVGGGCRKNKRATRKPSSSSAVVVPVAPSPAMPMSMMLHGGRHVETSGGLHLSFSGSTMQLP
SHTSAPDPLCSSLGLLDWKYDNVFSGSGGGTTFESANSEAAHFTGQGMMGIGGSRGGADCHALDALRYAASLGMGEHLALPFGAGARA
ERDHAVEMKPLSLEWCGEASRVPESSTISSLSGLGLWSGMISGAHHHHGSSAAI
>BdDOF13 Bradi3g29940.1
MASPFLAGSSSSSASSPLSYMIPSSSLALAAQGQGQGFLSCGVLAQQGPQGDAVGVGAPRQGGTGRHAGLPRPPPRDCPRCRSGNTKF
CYYNNYSRKQPRYLCRACRRHWTEGGTLRDVPVGGGRKSRRNNGAGSRKAAATKASASPSTADAAAAAGVDGSLVVPADVLRQMLFFQ
PAGFEGGYGIDMGAWQQQQMAAAAPNATKAPQGVGGEVGAGVEGTSASAADGVNCGAGMQFWSGGWQQMQDDMPGFDGTF
>BdDOf14 Bradi2g62380.1

MAPTAAGAGGDEAAINNNSSSKAMSMSERARLARVPQPEPGLNCPRCDSTNTKFCYFNNYSLTQPRHFCRACRRYWTRGGALRNVPVG
GGYRRHAKRSSKPKSSSTSAAAAASSSPAPATTTTCSTAAAAPQSSTLQSMFCNNSSNNNSSNMFDLGLSSSSFPFLQQQEQQQQWAQ
QMQQSFPFLQAMDMGMGQPMPLPAATMQGMFHLGLHTGAGDDNAHFGGGHHMAQLPPAPGKRSSEQQQDYAQISSGRHGGNGSMYGDH
QHVVNGGGGYAFYSSSSAAGN
>BdDOF15 Bradi1g07600.1
MIQELLGGTPMDQHHHHQQIKSATNAMAHHHVSSLPMVLQPISANPSPTSSSTSSRSSTQRSPSATSSPQGQGQQQGPEQAPLRCPRC
NSSNTKFCYYNNYNLTQPRHFCKTCRRYWTKGGALRNVPIGGGCRKPRPMPAPVAKQSTVVPAASCKSALSGTGIGMAPPPLGLGVGP
AMSSWAPQQLMALLNSTRAMQGLHHGGQGHGHGGGGNNVHRLLGLDTMGQLQVLPGHSPNNAMWPPVAHRPMPPPPPMHLDMAGPLGL
GLGGHSDLFSNLGLKPPSSSSLAPAASYYNDQLNAVVSNGGGGAGRPNAYESPSSYSCATTMASLPAASSTVSSGLTVGMDQQQPPVS
TSSSLAAQEMQYWNSGPAAMSMAAWPDLPALNGAFP
>BdDOF16 Bradi2g09720.1
MSDQMDSGIKLFGRVIPLAPEAASGPPEADAAAGSDHPPPPPPPPPPPPPELQTAAPAEEAEEEAHKDQHKETEDKEDSEMKVDVPPEK
ENRGIEGDAPREKEGNEMEVDAPQVNENAEPDSSSTLDHKKDTQDQINSAEDKADPKELNEKTANEESDQDKVLKKPDKIIPCPRCNS
MDTKFCYYNNYNINQPRHFCKNCQRYWTAGGTMRNVPVGAGRRKSKNSSLHYRQLLMAPDCMMGSRVDISNTVNPEVLASLPTTPTKS
TSRNETVLKFGPEVPLCESMASVLNIEEQNVINAGSVPIGEAREVNSCASSMTSHNGLPENAVHVDKNGAPVYCGGVGPMPPYYLGAP
FMYPWSIGWNNLPVMVPGGSMSESASPSESCSTSSAPWMNSPMMPGSRLPAPAFPYPLVPPTLWGCLPSWPATAWNAPWIGTNGCISP
STSSNSSCSGNGSPTLGKHSRDSNPLKDDKEEKSLWVPKTLRIDDPDEAAKSSIWATLGIKPGDPGVFKPFQFKSENKGQTTDVRPAC
VLQANPAAFSRSQSFQESS
>BdDOF17 Bradi2g50370.1
MAPLVGAFKLFGKVITPPVPAVAGEEEPQRLPGDGERDRTAAIKREAPAGDLEGEETKQQGQGGGAARRTQLQESAEARAAAAPLPCP
RCRSRDTKFCYFNNYNVNQPRHFCKACHRYWTAGGALRNVPVGAGRRKNRPLGPNVGANHHHHHRAPPAAGFNLAFPNAAASSPNSPS
PVYTDGHWQAGPDRRF
>BdDOF18 Bradi4g04260.1
MTERARLARVPLPEPGTLRCPRCDSANTKFCYFNNYSLSQPRHFCKACRRYWTRGGALRNVPVGGGCRRNTKRSNSKKSSRSNNSQSQ
SQATSSATSSCSTTTATSASTTSAAAMQALPHHLALQLDASLDGYHGHHAQLQFLPAFMQLQQQQQGMMMQHGGHGGGYGHFADGGVG
DVGSAGQQQLPDGFPRGMVASGLLAQLASIKMEEHGVANGGGGIGGGGGFQLGLQGAHEQYWPGSGGGGGGWPTEFLSGFSSSSSGNV
L
>BdDOF19 Bradi2g19930.1
MVEMGGGAAAAGFKLFGKVITRQQQQQPPTRASPDSSSSGGGRGSVEQLEEAARRARAAAGARPLPCPRCRSEDTKFCYFNNYNVNQP
RHFCRACHRYWTAGGAIRNVPVGSGRRKNRPVLPHADYQVRLAPGPASASASVEAPDMAPALGFIPAPEHGWSSLYVVPSPSPAAAAY
RGHGAEMEQCWWLVHAERPPLGSSDPAF
>BdDOF20 Bradi3g25670.1
MAECRGGDGLIKLFGKTIPVPEPVGALSKDLGHSGSSSTESGVQEITTDPSPQPEVVDAEDPAVDKGSQLQSGDDEAASEKEKLKKPD
KILPCPRCSSMDTKFCYFNNYNINQPRHFCKKCQRYWTAGGAMRNVPVGAGRRKNKNVLAASNFLQRVRAALPVDTFCSSPCPPVKTN
GTVLSFGHDASSTLDLAEHLKDNRIPIARSRNARDNPSMGSCSEVVSSNRNDKDQRNDITVEKSANGVQQQHPAGMNGGTMWPYGCTP
SPAAYYTSGIAIPIYPGAPGPGYWGCMVPGAWSLPWPVQCQPQALSSPTSAPSVSSAPSPLTLGKHPRDQADEGNRGHGNGNGKVWVP
KTIRIDNADEVARSSIRSLFGIKGDDRDEQISGTSGHKLATSVFEPKQHEAKMAKHAEAITSLPLLHHANPVALTRSVIFHEGS
>BdDOF21 Bradi1g15420.1
MAGDEAVAQQPRKGGTGTTAAQAQAPAAPGEAQQAGLRCPRCDSPNTKFCYYNNYSLSQPRHFCKTCRRYWTKGGALRNVPVGGGCRK
NKRSRSSSASSAAAASSRLSLNLPGPDHQHQQADLQQQQQHAAVRMGGGFRGVVDFHSGSMLPGPTSAVVSHGQYVPFGEWPAPGADV
NNNISHGMSGGGGGGGSSIASSIESLSYINQDLHWKLQQQRVATMFLGPPIAADAQAHHHHQFGAGAAFLQMETTTAVPAAATSWFMDS
SSSYAPAPLQPNSSSSPCRPPAAASCSNINSGRSTSGGGGGGDVDNATSNNNNCGGSDIPSWGDISTFAMLP
>BdDOF22 Bradi1g17410.1
MRITKENPSTQQEFDLGQINKSSLNNSTECENQAPNGDERTEQGSKSEAAKTEDDGSSRDKVLKKPDKILPCPRCKSMDTKFCYYNNY
NVHQPRHFCKGCQRYWTAGGSMRNIPVGAGRRKSKSSSSNCRSILTPGSSLAAPVGDASLIPFSVKGNEPVVTFGSDAPLCNSMASSL
RVEEQNKISNPASTAHPISGKNLTCPPPTTISDSQDTESVKGTVSGHQNGLAGDCNGVTPVHPIPCFPGPPFMYPWNPAWNGIAAMAT
PICPAQTESVKSSENGNGVNVQWNLPPMVPVPGFCGPPIPFPLMPPSVWPFVSPWPNGAWSAPWLGPGYSMPAAPPTSSITCSDSASP
VLGKHPRDSNLHGDEKSEKSLWIPKTLRIHDPDEAAKSSIWTTLGIEPGNRGMFRPFQSKSGSKEQMSDAARVMQANPAAQSRFQSFQ
ETT
>BdDOF23 Bradi3g51510.1
MEEMMMPAGNQNPNAANQNQNNQPPAPPSGPEGVQRAPAAAPAAAAAAGGAERKARPQKEKAINCPRCNSTNTKFCYYNNYSLQQPRY
FCKTCRRYWTEGGSLRNVPVGGGSRKNKRSSSSSSAAAAAVSTSAAAGTVPAANKNPKLMLQHEAGGGAHDLNLAFPHHHGRVLHPSE
FAASFPSLESSAVMAAANGGGRGGMHQGAFSAMELLRSTGCYVPLPQGMQLGAMPPEYGFALGPEFRMPAPPHQQQQQHHQQQQQVQN
MLGFSLDTGAGGGYGAGLQGAQESAASGRMLFPFEDLKPGVVSAAAGGATGGDQFEHSKADQGQGQGGNNSGGHETLGFWNNSMIGNG
GSNDAGGGGGGGSW
>BdDOF24 Bradi5g26240.1
MAPDAAATGKLPDQAPPLLAQGDHHQAGAGNGNGKQQQLECPRCGSGNTKFCYYNNYSTAQPRHFCRACRRYWTHGGSLRNVPVGGAC
RRRDAAASGNANKRRRASSSDSGYPSSSSAEPPSSAAQDQLPPPALTFPFLSDGTAFFLPPPPQYDISLGAGSAAAAFSWPLYDGGLG
AAPWDDGTLSGAAAGAATGAWGNVDEFTGLDLSWPPASGN
>BdDOF25 Bradi1g66600.1
MPPVGPTAAALASSSAAGNNEAAGQQQQQARPNSHSMTERARMARAPHPEPALKCPRCDSTNTKFCYYNNYSLSQPRHFCKACRRYWT
RGGALRSVPVGGGCRRNKRSSNKSSKPSSSSANRQQLPGGASASSSILPSTAPGSGVPSGAMIPPVGLGSSMAHHLPFLGSMQQHAPG
AGPNLGLAFSVGLPPLGIGMHQQQHMDGVDQSSFPLASGGAAATFGASLEQQWRVQQQQPHQQQFPFLELPPPPMYQLGALQANRAAG
SGAAAPSGMFTLGQTAASATATAARHERSVKQADDDSKGGQQEMSLQRQYMEALRQQGDHQAQGVWGGSGSATDDGNNGSGSWTMNLP
GFHSSSTGGHGGGLL
>BdDOF26 Bradi4g33000.1
MQEPGRRTAPPFAGVDLRRPKGYQALAQAEACPRCESRDTKFCYYNNYNTTQPRHYCRSCRRYWTKGGSLRNVPVGGASRNNSSSSSS
SSSSPPKRTKNSSSNSKRRRVVPEPEHEPVRTDASAPAATTEAVRPVTTEDAAPDDPAVETEPSVKLGVGGGLADAGGKEPSPFEWPS
GCDDLAISYWGTGVLADADPAMFLNLP
>BdDOF27 Bradi2g46300.1
MANLPTQAATADASGFKLFGKVIQPDAAAQHDASASITASTSTEESTPPPPPPPPPPLPPSPPPLQAPAGGEPLPCPRCGSRETKFCY
FNNYNVRQPRHLCRACRRYWTAGGALRRVASASPGRRRPRPNAARSAAAAASASASASEGAAESVDSLS
