# Supplementary material for: The family of DOF transcription factors in Brachypodium distachyon: phylogenetic comparison with rice and barley DOFs and expression profiling
Source: BMC Plant Biol. 2012 Nov 5;12:202. doi: 10.1186/1471-2229-12-202 (PMC3579746; doi:10.1186/1471-2229-12-202)
Supplement: Additional file 2 — Distribution of the conserved motifs along the BdDOF transcription factors clustered in Figure2. Motifs identified by means of the MEME software using the deduced amino acid sequences of the 27 Brachypodium BdDof genes represented in Figure 2. Position of the identified motifs is relative to the DOF domain. Multilevel consensus sequences for the MEME defined motifs are listed in Additional file 3. [file 1471-2229-12-202-S2.pdf]

Additional file 2 – Distribution of the conserved motifs along the BdDOF transcription factors clustered in Figure 2.

Motifs identified by means of the MEME software using the deduced amino acid sequences of the 27 Brachypodium *BdDof* genes represented in Figure 2. Position of the identified motifs is relative to the DOF domain. Multilevel consensus sequences for the MEME defined motifs are listed in Additional file 3.

CLUSTER *A*	BdDOF4	3		21	DOF	16	13	17	22		2	4
	BdDOF22			21	DOF	16	13	17	22		2	4
	BdDPF20	3	24	21	DOF	27	19	18	22	9	2	4
	BdDOF11	3	24	21	DOF	27	19	18		9	2	4
	BdDOF16	3		21	DOF	16	13	17	22		2	4
	BdDOF17	3			DOF							
	BdDOF19	3			DOF							
	BdDOF27	3			DOF							
CLUSTER B	BdDOF7				DOF							
	BdDOF26			14	DOF							23
	BdDOF3			14	DOF							23
CLUSTER C	BdDOF8				DOF							
	BdDOF15				DOF							
	BdDOF21				DOF							
	BdDOF12				DOF							
	BdDOF2	20			DOF		5	12			15	11
	BdDOF23	20			DOF		5	12			15	11
	BdDOF10				DOF			10			7	8
	BdDOF9				DOF			10			7	8
	BdDOF25			6	DOF							
	BdDOF14			6	DOF							
	BdDOF5			6	DOF			26				
	BdDOF18			6	DOF			26			25	
	BdDOF1			6	DOF						25	
	BdDOF6				DOF							
CLUSTER *D*	BdDOF24				DOF							
	BdDOF13				DOF							
