# Supplementary material for: The family of DOF transcription factors in Brachypodium distachyon: phylogenetic comparison with rice and barley DOFs and expression profiling
Source: BMC Plant Biol. 2012 Nov 5;12:202. doi: 10.1186/1471-2229-12-202 (PMC3579746; doi:10.1186/1471-2229-12-202)
Supplement: Additional file 3 — Group and sub-group of consensus specific conserved amino acid motifs. Numbers correspond to the motifs described in Additional file 2. Consensus sequences obtained from the analysis of the 27 Brachypodium DOF proteins with the MEME software. Motif 1 corresponds to the DOF DNA-binding domain. [file 1471-2229-12-202-S3.pdf]

Additional file 3 - Group and sub-group of consensus specific conserved amino acid motifs.

Numbers correspond to the motifs described in Additional file 2. Consensus sequences obtained from the analysis of the 27 Brachypodium DOF proteins with the MEME software. Motif 1 corresponds to the DOF DNA-binding domain.

Motif	E-value	Multilevel consensous sequence
**1**	7.5e-1149	**CPRC[DN]Sx[ND]TKFCY[YF]NNY[SN]LSQPR[HY]FC[KR]ACRRYWT[AKR]GG[AS]LRNVPVGGG[CR]R[KR]**
**2**	3.8e-062	**[DEH][EDGT][KN][REGS][EN][GKN][KST][LV]W[VI]PKT[ILR]RI[DH][DGN][APT][DA]E[AV][AT][KR]SSI[WLR][SADT][TL][LFI]GI[KEN][GPH][DG][DEKN][RNPV][GD][ELMTV][FDQ][KDIR][PSG][FGR][QGT][SCFR][KG]**
**3**	1.1e-024	**[FI]KLFG[KR][VT]I[PT]**
**4**	1.0e-010	**[VFLT][LHM][QHK][AG]NP[AV]A[FLQ][ST]R[SF][QV][SIT]F[QH]E**
**5**	1.8e+000	**MELLRSTGCY[MV]**
**6**	7.3e-014	**M[TS][ED]RAR[LM]A[RK][VAI]P[LAHQ]PE**
**7**	9.2e-001	**D[AG]S[MT]IDLA[LM]LYSKFL[NS][HN]Q**
**8**	2.4e+001	**EL[AN]F[SV][MV]DQSC[FY]D**
**9**	6.2e-006	**Y[FY]TS[GS][IV]AIP[FI]YP[AG]A[AP][AG][AP][AG]YWG[CY]MVPGAW[NS][LT]PWP[PV]Q[CS]Q[PS]Q**
**10**	2.3e+001	**RPDM[LV]LEGMVGN**
**11**	2.1e-001	**RMLFPFEDLK**
**12**	4.7e-003	**KNPKL[AM][HL][HQ]HE[AG]GG[AG][AQ]HDLNL[AS]FPHH**
**13**	4.6e-004	**W[GPS][CF][][PS][GPS]WP[NA][GT][AT]W[SN][AS]PW**
**14**	1.8e+001	**FAGVDLRRPKGY**
**15**	1.1e+002	**FPSLESS[AS][IV]**
**16**	1.3e-010	**[ITV][KS][EGR]NE[TP][VA][KLV][EKT]FG[SP][ED][VA]P[]C[NE]SMA[ST][VS]L[DNR][INV][EG]EQ[NK][GKV]I**
**17**	3.6e-005	**[YC][FPY][LNP]G[PA][PA][FL][MV][YF]PW[SN][PI][GA]WN[GNS][IL][AP][VA]M**
**18**	1.7e+000	**MNGGTMWP[FY][GS]C**
**19**	7.5e+000	**C[PT]P[IV]KTN[AG]TVLSFG[HS]D**
**20**	5.9e+001	**MEEM[LM]M[GP][AT][GT]NQ**
**21**	2.3e-024	**[KES][TEG][EADQ][DSN][DE][EAGT][ASL][DSN][QER][DK][KE][VK]LKKPD[KI]I[LIV]P**
**22**	1.0e-002	**[IS][TSV][CS]S[ADEG][NPS][AGNS][SP][PL][TV]LGKH[PS]R[DE]**
**23**	2.9e+002	**FEWP[PS][AG][CF]D**
**24**	1.1e+003	**KD[LQ][GQ]HSGSS[ST][AT]E[PS][GK]VQE[IN][IT]**
**25**	1.2e+003	**L[IL][AT]QLAS[IV]KME**
**26**	1.0e+003	**[RY]H[GP]HH[AQ]Q[LQ]Q[FV]**
**27**	4.0e+003	**K[NS][IV][LS]AAS[HN]FLQR[IV]RA[AT]LP[GV]D**
