# Supplementary material for: The family of DOF transcription factors in Brachypodium distachyon: phylogenetic comparison with rice and barley DOFs and expression profiling
Source: BMC Plant Biol. 2012 Nov 5;12:202. doi: 10.1186/1471-2229-12-202 (PMC3579746; doi:10.1186/1471-2229-12-202)
Supplement: Additional file 4 — Primer sequences used for RT-qPCR analyses, amplicon length and PCR efficiency. The corresponding RT-PCR efficiency (E) of one cycle in the exponential phase has been calculated according to the ecuation E=10(−1/slope). [file 1471-2229-12-202-S4.pdf]

Additional file 4– Primer sequences used for RT-qPCR analyses, amplicon length and PCR efficiency.

The corresponding primer PCR efficiency (E) of one cycle in the exponential phase has been calculated according to the ecuation $E=10^{(-1/slope)}$

Brachypodium Dof genes	Primer sense	Primer antisense	Amplicon size (bp)	Tm (^{0}C)	Slope	E*
BdDof1	CATGCGGTTTGTGCTATGTC	CGTGGTTCTTCCTTTGGTTC	128	78.4	-3.14	2.08
BdDof2	ATGTGGAGACGTGGAAGGTC	CAGCCTTCCGCTTGTAGTTG	177	81.1	-3.06	2.13
BdDof3	TCATCATCAGTTCGGAGCAG	TGTCCATGAACCAAGACGTG	89	78.6	-3.48	1.94
BdDof4	TCGATCTCCATGACTCCATC	GATCCATTGTTCCGCAGTTC	146	73.5	-3.30	2.01
BdDof5	GCACCGGTGTTACTGAATTAGG	CGAACGACACAAGACCATACC	100	70.8	-3.57	1.90
BdDof6	GGAGGGAATTGGAGAAGTTG	AATCCCCTACCTTTGCCATC	94	71.5	-3.11	2.09
BdDof7	ATCGATGGAGGGGTGTTATG	CATCCACACCATGAGCAAAC	79	68.5	-3.54	1.91
BdDof8	GGGAAGAGATCTTCAGAGCAG	CTCCATACATGCTGCCATTG	82	74.2	-3.59	1.90
BdDof9	GCGGAAATGATAGGGGATAG	CAAACCCAGCCAAAAGAGAG	103	72.1	-3.53	1.92
BdDof10	GGGCCAACTTTAGGAGACTTG	CACAAAGCTGAAACCACTGC	99	73.4	-3.02	2.14
BdDof11	AAGTACGAGCCGTTCGATTC	TTGAGAGCCCAACACTTTGC	108	79.87	-3.18	2.06
BdDof12	AAAGAGGAGAGGAGAGATGAGG	CACTAGGATCACCGTATGACACC	109	68	-3.01	2.14
BdDof13	AAAGCAGGCTTTGAGACGAG	CCGTGTATGCAAGAACAAGC	110	72.4	-3.64	1.88
BdDof14	TGCGGATGTAGGTTTCTGTG	TGTAGATTTCAGGGCCAAGC	129	74.7	-3.59	1.90
BdDof15	GCTCATGCTGTGCTGTTTTG	CGACACGATGGTTAGCTTTG	109	74.4	-3.57	1.90
BdDof16	CAATGATCTGGCAACGTAGG	CTGGCAATCTGACCATGAAC	126	74.1	-3.58	1.90
BdDof17	GCTCACTCCTGTTTGCTTGTC	TCTTTGCAAGCTCCACTGC	145	71.6	-3.56	1.91
BdDof18	TATTTCCTACCGCCTCCATC	AACTCAAGATGACGCCCAAC	116	72.1	-3.43	1.95
BdDof19	TTCATGCTGCCTTCTGCTTC	GTACACTCCATTTCCCCTTACC	101	68.2	-3.56	1.91
BdDof20	CCTGGTCCTGTGTAGATCAATG	GCTGATGCACAAAACACCTC	75	68.4	-3.53	1.92
BdDof21	GATGCCCCTTTTCTGTTCTG	GCGATGCAACCTTACAAACC	127	70.4	-3.55	1.91
BdDof22	GCTCGATCGCTTTTGATTACC	TCATGGATCCAAACCCTAGC	92	72.5	-3.53	1.92
BdDof23	TCCGGATTCAAGCTCTTCG	TCTTCCGTGGAGGTGGAG	95	80	-3.33	1.99
BdDof24	ATATGAGAGCAGCAGCATGG	GCAAATGCAAGGTAGAGCAAC	110	71.4	-3.50	1.93
BdDof25	ACCACCAGCAGATCAAGAGC	ACGACGTCGAGGAAGATGTC	117	80.1	-3.02	2.14
BdDof26	CGGATGGTGTCAACTGTGG	GGCATGTCATCTTGCATCTG	73	75.9	-3.16	2.07
BdDof27	GCTCATCCGATGCATTTCTG	CATAGTATCGGACGGGGAAG	114	78.4	-3.15	2.08
BdGAPDH	CTCCCGCTATTTCGTTTGTC	TGAAGATGTTGGAGCTGACG	76	71.5	-3.07	2.11
