# Supplementary material for: The family of DOF transcription factors in Brachypodium distachyon: phylogenetic comparison with rice and barley DOFs and expression profiling
Source: BMC Plant Biol. 2012 Nov 5;12:202. doi: 10.1186/1471-2229-12-202 (PMC3579746; doi:10.1186/1471-2229-12-202)
Supplement: Additional file 5 — Expression patterns of the BdDof genes in varios organs of the Brachypodium distachyon plants. The numbers indicate the relative expresion levels standardized to BdGAPDH (mean ± standard error). [file 1471-2229-12-202-S5.pdf]

Additional file 5. Expression patterns of the *BdDof* genes in varios organs of the *Brachypodium distachyon plants*. The numbers indicate the relative expresion levels standardized to BdGAPDH (mean + standard error)

Brachypodium dof genes	Leaves	Roots	Spikes	Developing Seeds	Dry Embryo	Germinating Embryos	Germinating Aleurones
	Mean±SE	Mean±SE	Mean±SE	Mean±SE	Mean±SE	Mean±SE	Mean±SE
BdDof2	145.19±8.12	36.46±3.44	24.61±0.55	32.65±2.58	6.92±0.80	1.68±0.05	0.85±0.06
BdDof3	930.30±24.95	78.03±3.99	320.96±7.17	412.45±10.38	26.63±0.82	19.98±1.20	30.21±1.88
BdDof4	12.20±0.61	3.48±0.07	13.70±1.17	61.80±4.73	35.19±2.05	6.00±0.36	21.03±2.02
BdDof8	830.90±25.32	0.48±0.25	43.49±2.21	37.26±2.35	1.59±0.37	1.08±0.07	15.61±1.98
BdDof16	490.97±19.90	32.95±0.21	11.27±0.44	10.56±1.63	59.77±4.68	9.45±0.83	27.52±3.36
BdDof17	12.19±1.34	1.72±0.61	6.88±0.06	19.05±1.70	52.16±3.37	3.74±0.14	35.97±1.53
BdDof22	306.55±13.36	7.76±0.97	6.38±0.00	22.44±3.16	47.47±3.94	7.41±0.36	34.92±2.02
BdDof23	302.15±11.21	99.96±10.69	19.62±1.70	39.00±2.27	16.50±0.34	23.68±1.23	7.92±0.30
BdDof24	12.83±0.36	2.55±0.32	2.47±0.20	10.99±1.25	5.43±0.12	56.14±2.18	116.57±16.29
BdDof1	113.78±11.98	16.60±0.48	3.43±0.11	8.11±0.48	1.83±0.26	2.64±0.23	1.78±0.40
BdDof5	43.47±1.37	22.92±3.28	7.05±0.35	12.86±0.84	5.22±0.02	17.44±0.69	7.76±1.79
BdDof6	84.84±5.43	22.09±1.46	16.19±0.48	18.58±1.43	2.09±0.01	4.17±0.09	1.47±0.12
BdDof7	1.58±0.02	0.38±0.02	0.34±0.03	1.38±0.02	0.66±0.02	0.78±0.01	1.69±0.07
BdDof9	0.00±0.00	0.14±0.05	0.26±0.01	0.31±0.05	0.00±0.00	0.35±0.01	1.76±0.03
BdDof10	0.16±0.11	0.00±0.00	1.74±0.06	1.44±0.22	0.16±0.01	3.48±0.07	11.02±0.79
BdDof11	944.62±44.13	6.69±0.29	8.05±0.34	7.41±0.49	22.69±0.88	2.24±0.20	5.92±0.97
BdDof12	54.69±2.38	0.95±0.11	1.65±0.09	1.31±0.02	0.17±0.01	2.21±0.41	0.97±0.02
BdDof13	0.06±0.01	0.26±0.01	0.08±0.01	0.21±0.03	0.14±0.01	0.00±0.00	0.00±0.00
BdDof14	28.31±1.37	29.90±2.50	1.02±0.23	14.43±0.59	2.55±0.04	5.73±0.57	1.04±0.01
BdDof15	0.88±0.02	0.10±0.00	0.18±0.03	0.51±0.02	0.34±0.02	2.34±0.21	2.58±0.42
BdDof18	110.33±3.83	5.28±0.18	5.07±0.05	12.01±1.16	1.60±0.05	6.54±0.023	1.11±0.12
BdDof19	7.86±0.45	5.44±0.30	1.03±0.24	5.21±0.17	1.46±0.08	1.47±0.11	1.17±0.18
BdDof20	26.10±2.37	3.07±0.46	3.01±0.09	2.05±0.68	5.85±0.36	0.85±0.01	1.48±0.43
BdDof21	23.27±0.40	0.00±0.00	0.44±0.03	0.55±0.09	0.16±0.01	0.23±0.02	0.66±0.38
BdDof25	69.85±2.72	29.56±1.45	7.75±0.46	7.28±0.14	0.64±0.01	2.91±0.35	1.30±0.02
BdDof26	21.94±0.82	8.89±0.40	9.50±0.43	4.57±1.01	0.96±0.18	1.66±0.21	11.79±1.80
BdDof27	205.22±10.68	11.76±0.02	20.04±0.83	6.49±0.07	20.31±1.24	24.10±0.91	0.86±0.07
