# Supplementary material for: The family of DOF transcription factors in Brachypodium distachyon: phylogenetic comparison with rice and barley DOFs and expression profiling
Source: BMC Plant Biol. 2012 Nov 5;12:202. doi: 10.1186/1471-2229-12-202 (PMC3579746; doi:10.1186/1471-2229-12-202)

Additional file 7 – Joined phylogenetic tree of the Brachypodium, barley, rice and the most important DOF genes characterized so far.

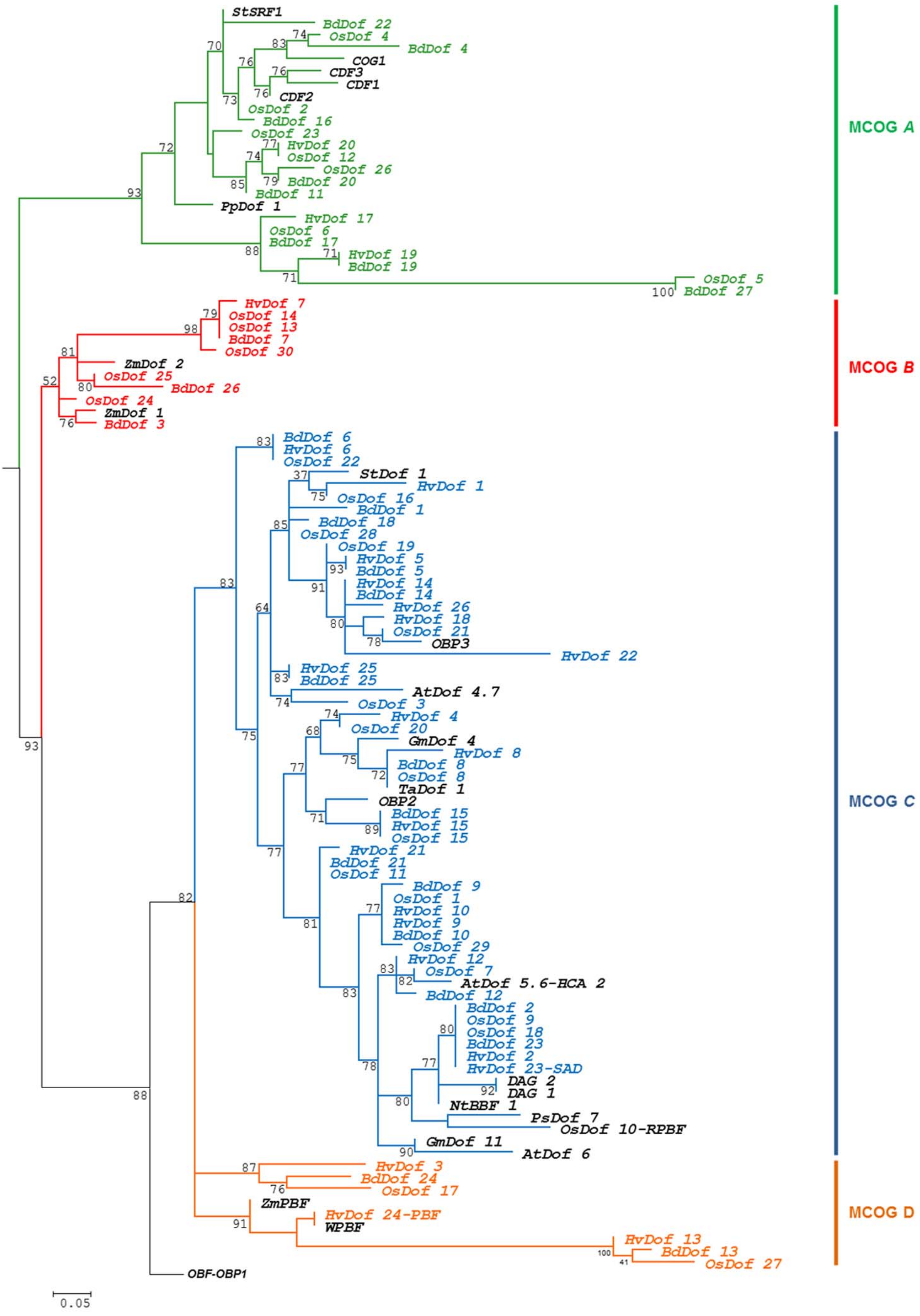

Supplement: Additional file 7 — BdGAPDHexpression in different organs (A) and at developmental stage of maturating (B) and germinating (C) seed. [file 1471-2229-12-202-S7.pdf]
